# Supplementary material for: MKL/SRF and Bcl6 mutual transcriptional repression safeguards the fate and positioning of neocortical progenitor cells mediated by RhoA
Source: Sci Adv. 2023 Nov 15;9(46):eadd0676. doi: 10.1126/sciadv.add0676 (PMC10651131; doi:10.1126/sciadv.add0676)
Supplement: 20231115-1 [file sciadv.add0676.v1.pdf]

DEVELOPMENTAL NEUROSCIENCE

MKL/SRF and Bcl6 mutual transcriptional repression safeguards the fate and positioning of neocortical progenitor cells mediated by RhoA

Alexia Cossard, Kirsten Stam†, Aurore Smets†, Yves Jossin*

Copyright © 2023 The Authors, some rights reserved; exclusive licensee American Association for the Advancement of Science. No claim to original U.S. Government Works. Distributed under a Creative Commons Attribution License 4.0 (CC BY).

During embryogenesis, multiple intricate and intertwined cellular signaling pathways coordinate cell behavior. Their slightest alterations can have dramatic consequences for the cells and the organs they form. The transcriptional repressor Bcl6 was recently found as important for brain development. However, its regulation and integration with other signals is unknown. Using in vivo functional approaches combined with molecular mechanistic analysis, we identified a reciprocal regulatory loop between B cell lymphoma 6 (Bcl6) and the RhoA-regulated transcriptional complex megakaryoblastic leukemia/serum response factor (MKL/SRF). We show that Bcl6 physically interacts with MKL/SRF, resulting in a down-regulation of the transcriptional activity of both Bcl6 and MKL/SRF. This molecular cross-talk is essential for the control of proliferation, neurogenesis, and spatial positioning of neural progenitors. Overall, our data highlight a regulatory mechanism that controls neuronal production and neocortical development and reveal an MKL/SRF and Bcl6 interaction that may have broader implications in other physiological functions and in diseases.

INTRODUCTION

The mammalian cerebral cortex plays key roles in cognition, emotions, and sensory and motor functions. These tasks rely on adequate activities of neural circuits. This connectivity depends on the ability of proliferative cells of the neuroepithelium to produce an array of neurons and glial cells that carry out specialized functions in mature neural networks (*1*). The ventricular zone (VZ) and subventricular zone (sVZ) are the proliferative regions of the neocortex respectively populated by the radial glia cells (RGCs) and basal progenitors (BPs). Under the influence of local environmental cues, RGCs, the neural stem cells (NSCs) of the neocortex, progressively lose their proliferative potential as development proceeds (*2*, *3*). They either divide symmetrically to expand and produce two RGCs or asymmetrically to give rise to another RGC and either a neuron (direct neurogenesis) or a BP that will produce several neurons (indirect neurogenesis) (*4*–*6*). During neurogenesis, symmetric and asymmetric divisions coexist at the VZ (*7*). RGCs undergo an interkinetic nuclear movement (IKNM), which is an oscillation of the position of their nuclei during the cell cycle (*8*). The function of this movement is not well understood, although it is believed to be important for the regulation of cell fate decision (*9*).

Together, RGCs and BPs sequentially produce the deep- and superficial-layer excitatory glutamatergic neurons that account for approximately 80% of all cortical neurons (*3*, *10*–*14*). The establishment of BPs and the consequent increase in the neuron numbers per radial glial cell is thought to be at the origin of the evolutionary expansion of the mammalian cerebral cortex (*15*–*17*). A proper positioning of BPs in the sVZ seems to be important for a correct migration of the neurons they produce (*18*). However, while the migration of neurons is extensively studied, little is known about the migration of BPs out of the VZ to incorporate in and form the sVZ.

A tight and coordinated control of proliferation, neurogenesis, and migration has evolved to ensure the correct production of neurons at the right time and the right place. Subtle alterations in the balance between RGC self-renewal or differentiation during development, as well as defects in neural cell migration and positioning, can result in dramatic differences in neocortical size at the origin of human brain evolution and of developmental brain malformations such as microcephaly, Down syndrome, autism spectrum disorders, lissencephalies, or periventricular heterotopias (*19*–*28*). However, our knowledge of the pathways regulating these processes in the developing cerebral cortex and their interactions remains incomplete.

The Rho subfamily is composed of three members (RhoA, B, and C). RhoA expression within the embryonic neocortex is higher at the VZ and sVZ, while RhoB is mainly expressed in the cortical plate, and RhoC is barely detectable (*29*). Loss of RhoB or RhoC is not associated with any apparent consequences on embryonic development (*30*, *31*). On the other hand, conditional knockout of RhoA in early NSCs in the mouse cortex or spinal cord both result in loss of cell adhesion in the germinal zone, causing a dramatic disorganization of the tissue, while proliferation is increased in the cortex but decreased in the spinal cord (*32*–*34*). RhoA functions may therefore be tissue specific. In addition, it is known that instructive factors and progenitor competence change over time (*35*). Differences in RhoA functions during early and late neurogenic phases in the developing cortex could therefore exist, underscoring the influence of the interplay between environmental cues and cell-intrinsic information on cell fate and cell behavior. Recent data demonstrated the importance of primate-specific RhoA regulators in cortical organization, suggesting a role in the control of RhoA in human-specific evolutionary development and neuropsychiatric disorders (*36*). Nevertheless, little is known about the role of RhoA in neurogenesis and the mechanisms involved. Even less is

Laboratory of Mammalian Development and Cell Biology, Institute of Neuroscience, Université Catholique de Louvain, Brussels 1200, Belgium.
*Corresponding author. Email: yves.jossin@uclouvain.be
†These authors contributed equally to this work.

known about its functional interactions with other regulatory pathways.

Dynamic and coordinated regulation of gene expression is crucial during organogenesis. This regulation depends on the balance between activating and repressive transcription factors and regulators that act in trans to control gene transcription (*37*). Well-known effectors downstream of RhoA are the family of transcription coactivators myocardin-related transcription factors, including myocardin and megakaryoblastic leukemia 1 and 2 (MKL1 and MKL2). RhoA regulates their activity through the modulation of the actin cytoskeleton. In turn, they regulate the transactivation activity of the serum response factor (SRF) transcription factor that binds to SRF response element (SRF-RE) DNA sequences (*38*). The function of SRF during neocortical development is still unclear. *Srf*-null mice are early embryonic lethal (*39*). While some studies demonstrated the involvement of SRF in projection neurons' radial migration (*40*–*42*), *Srf-Nestin*-cKO mice showed no defects in neurogenesis and radial migration in the neocortex (*43*). However, the absence of phenotype in these mice could be due to the rate of *Nestin-cre*–driven recombination that only reaches sufficiently high levels during late embryonic period when floxed mice are crossed with the *Nestin-cre* line from the Jackson

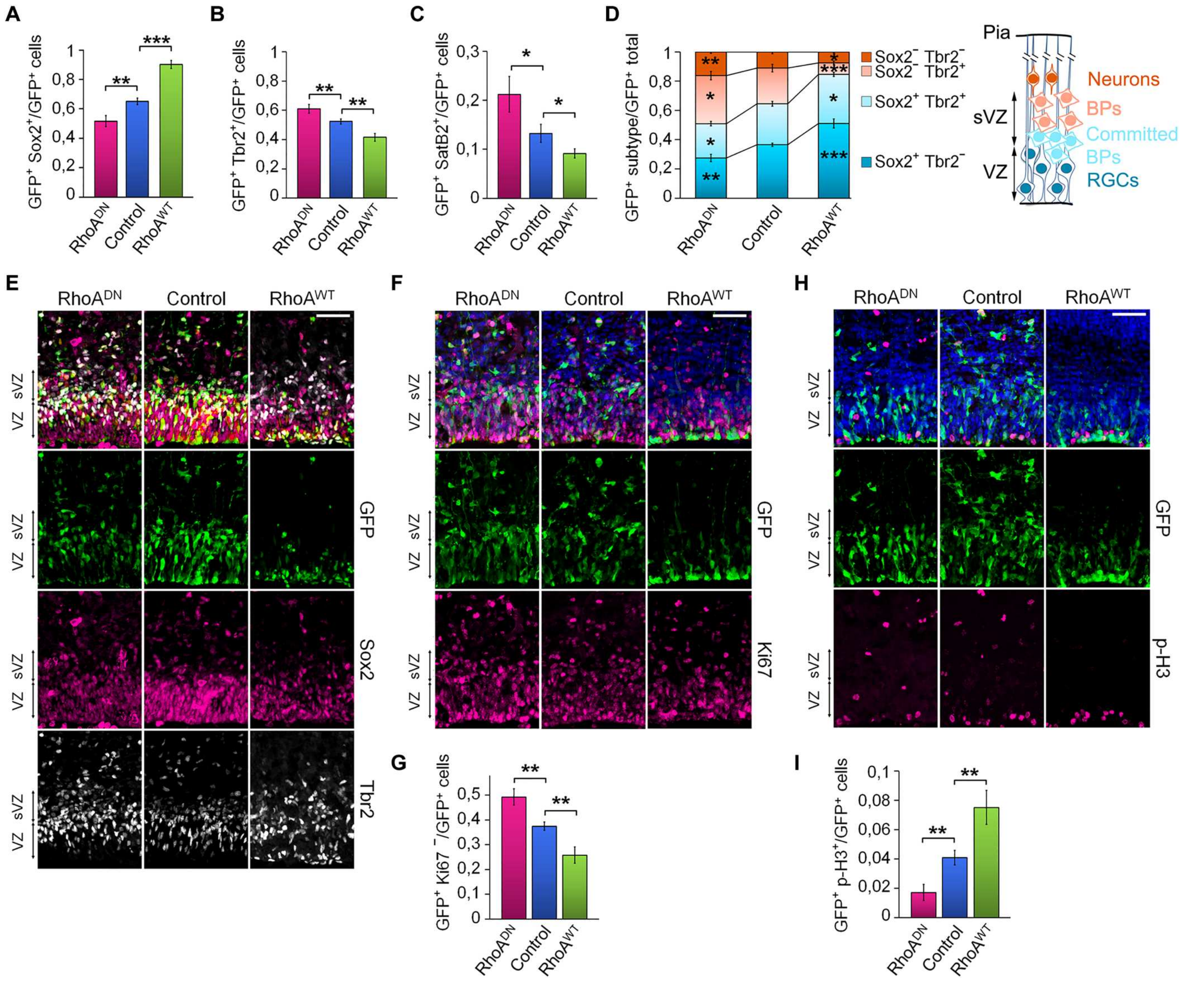

Fig. 1. RhoA reduces neurogenesis and maintains the neural progenitor pool size. Embryonic brains were in utero electroporated at E15.5 and processed 23 hours later for immunohistological labeling. (**A** to **C**, **G**, and **I**) Quantification of double staining for (A) GFP$^+$Sox2$^+$, (B) GFP$^+$Tbr2$^+$, (C) GFP$^+$SatB2$^+$, (G) GFP$^+$Ki67$^-$ (cell cycle exit), and (I) GFP$^+$p-H3$^+$ (mitotic index) cells. (**D**) Quantification of the triple-staining GFP$^+$Sox2$^+$ Tbr2$^-$ (RGCs), GFP$^+$Sox2$^+$Tbr2$^+$ (committed BPs), GFP$^+$Sox2$^-$Tbr2$^+$ (BPs), and GFP$^+$Sox2$^-$Tbr2$^-$ (neurons) cells. (**E**, **F**, and **H**) Coronal sections of E16.5 cerebral cortices electroporated at E15.5 for the expression of NLS-GFP alone (control), dominant-negative form of RhoA (RhoA^{DN}), or wild-type RhoA (RhoA^{WT}), along with NLS-GFP and immunostained for (E) Sox2 and Tbr2, (F) Ki67, or (H) p-H3. (A) Control n = 19 of 13 IUE (n = 19//13); RhoA^{WT} n = 8//6; RhoA^{DN} n = 9//9; (B) Control n = 21//14; RhoA^{WT} n = 8//6; RhoA^{DN} n = 10//8; (C) Control n = 4//3; RhoA^{WT} n = 4//3; RhoA^{DN} n = 4//4; (D) Control n = 9//5; RhoA^{WT} n = 7//6; RhoA^{DN} n = 8//7; (G) Control n = 12//9; RhoA^{WT} n = 9//7; RhoA^{DN} n = 11//6; (I) Control n = 7//4; RhoA^{WT} n = 7//6; RhoA^{DN} n = 9//6. Error bars, SEM. ***P < 0.001, **P < 0.01, *P < 0.05. Scale bar, 50 μm.

Laboratory (*44*). This leaves open the question of an involvement of SRF in neocortical neurogenesis.

The transcription repressor B cell lymphoma 6 (Bcl6) is extensively studied for its importance in B cell development and oncogenesis but has been recently identified as a proneurogenic factor during the embryonic development of the neocortex (*45*). While some of its downstream targets during neocortical development have been identified (*45*, *46*), its upstream regulation and its integration with other regulatory signals are unknown.

Here, we found that RhoA, the MKL1/2 and SRF transcription factor complex, and the transcription repressor Bcl6 interact functionally to safeguard neurogenesis and cell positioning in vivo. A tight control of the RhoA pathway is necessary to regulate neurogenesis, the IKNM, and cell cycle length of RGCs, as well as the positioning of BPs in the developing mammalian neocortex. These functions depend on the cross-talk between the MKL/SRF complex and Bcl6. Mechanistically, Bcl6 inhibits the RhoA-mediated MKL/SRF transcriptional activity by physically interacting with SRF, observed in a tripartite complex with MKL1, and preventing SRF homodimerization. Similarly, the interaction of MKL/SRF with Bcl6 hinders Bcl6 dimer formation, reducing Bcl6 transcriptional repression activity and its effect on neurogenesis. Overall, these findings reveal that RhoA, MKL, SRF, and Bcl6 safeguard neocortical development by establishing appropriate progenitors positioning and by adjusting RGCs neurogenic choice to ensure proper neuronal output.

RESULTS

RhoA maintains the neural progenitor pool size

We considered that RhoA may have more subtle effects on the fate choice of RGCs that are not evident when RhoA is conditionally deleted throughout the VZ. Therefore, we used in utero electroporation (IUE) to manipulate RhoA activity [expression of a wild-type (RhoA^{WT}) or a dominant negative form of RhoA (RhoA^{DN}) or RhoA knockdown using specific small interfering RNA (siRNA)] in a subset of RGCs at embryonic day 15.5 (E15.5) in a murine model (*47*, *48*). At this stage of mid-neurogenesis, 76% of RGCs normally undergo asymmetric division to produce BPs (60%) or to make neurons directly (16%), while 15 to 23% symmetrically divide to generate two RGCs (*6*, *12*, *14*, *49*, *50*). Electroporated brains were dissected and labeled 23 hours later to analyze the altered cells and their progeny using specific markers for electroporated cells [green fluorescent protein (GFP)], RGCs (Sox2), BPs (Tbr2), and neurons (Satb2). RhoA inhibition reduced the percentage of GFP$^+$Sox2$^+$ and increased the proportion of GFP$^+$Tbr2$^+$ and GFP$^+$Satb2$^+$ (Fig. 1, A to C). RhoA gain of function (GoF) had the exact opposite effect, showing more GFP$^+$Sox2$^+$ and a decrease in GFP$^+$Tbr2$^+$ and GFP$^+$Satb2$^+$ (Fig. 1, A to C). However, these measurements did not consider cells positive for both Sox2 and Tbr2, corresponding to newly born committed BPs. Thus, we performed a triple staining to specifically label the four populations of cells observed at that stage: RGCs (GFP$^+$Sox2$^+$Tbr2$^-$), newly born committed BPs migrating away from the VZ (GFP$^+$Sox2$^+$Tbr2$^+$), differentiated BPs (GFP$^+$Tbr2$^+$Sox2$^-$), and newly born migrating neurons (GFP$^+$Tbr2$^-$Sox2$^-$ or GFP$^+$Satb2$^+$). We found that inhibiting RhoA reduced the proportion of RGCs and committed BPs and increased differentiated BPs and neurons (Fig. 1, D and E). RhoA GoF had the exact opposite effect. The IUE technique specifically targets RGCs undergoing S and G_2-M phases (*51*, *52*). At E15.5, the cell cycle lengths of RGCs and BPs are respectively about 18.5 and 26.5 hours (*53*, *54*). As we were collecting brains 23 hours after electroporation, it is most likely that the effect on the generation of postmitotic migrating neurons arises from a function of RhoA in RGCs since BPs could not yet perform an entire division cycle. Accordingly, results suggest that RhoA affects both direct neurogenesis from RGCs and indirect neurogenesis through the production of BPs. It did not disturb the production of glial precursors (Olig2$^+$ cells), which was negligible at this time of development (fig. S1A). Additional staining for the proliferation marker Ki67 showed an increased cell cycle exit induced by RhoA inhibition and reduced by RhoA GoF (Fig. 1, F and G). A positive influence of RhoA activity on the mitotic index was observed as well (Fig. 1, H and I). The specific knockdown of *RhoA* using siRNA resulted in the same phenotype as when expressing RhoA^{DN} (fig. S1, B to F). Together, these data suggest that RhoA inhibition increases direct and indirect neurogenesis along with RGC depletion, whereas RhoA GoF reduces neurogenesis and maintains the RGC pool size.

RhoA regulates the position of RGCs and BPs

To test whether RhoA regulates migration of the different populations derived from the VZ, we shifted our attention to the radial distribution of the electroporated cells. RhoA inhibition resulted in an overall increased distance of the GFP$^+$ cells away from the apical surface, with more cells located in the basal half of the VZ and the apical half of the sVZ, when compared to the control GFP$^+$ cells (Fig. 2, A and B). The specific knockdown of *RhoA* using siRNA resulted in the same phenotype as when expressing RhoA^{DN} (fig. S1, G and H). Conversely, RhoA GoF resulted in a shorter distance with accumulation of cells at the surface of the ventricle (Fig. 2, A and B). Marker analysis of control brains showed that, as expected, most of the cell bodies of GFP$^+$Sox2$^+$Tbr2$^-$ RGCs remained at the VZ (Figs. 1E and 2C). Newly born GFP$^+$Sox2$^+$Tbr2$^+$ committed BPs migrating away from the VZ were located at the basal side of the VZ and the apical side of the sVZ (Figs. 1E and 2D). The more differentiated GFP$^+$Sox2$^-$Tbr2$^+$ BPs were spread into the sVZ with a peak at its center (Figs. 1E and 2E). Last, the few observed GFP$^+$Sox2$^-$ Tbr2$^-$ newly born neurons were mainly located in the sVZ and the intermediate zone (IZ) (Figs. 1E and 2F). However, inhibiting RhoA increased the distance of RGC bodies away from the ventricular surface and led to their accumulation within the basal half of the VZ and the apical side of the sVZ (Figs. 1E and 2C and fig. S1I). Time-lapse videomicroscopy showed the expected IKNM from control RGCs located in the VZ. Only cells with a visible apical process attached to the apical surface were measured. A substantial proportion of nuclei from RhoA-inhibited RGCs did not perform the IKNM and remained at a basal position (movies S1 and S2 and Fig. 2, G and H).

Migrating committed BPs were still distributed within the basal side of the VZ and the apical side of the sVZ. However, they showed a sharper relative distribution toward the lower bins due to a fraction of cells that did not migrate as far as their control counterparts. Similarly, some differentiated BPs were not located as far as in the control condition, and the peak of their distribution shifted more basally within the sVZ (Figs. 1E and 2, D and E, and fig. S1, J and K). While the location of the neuronal population is more difficult to assess because of its small number, fewer RhoA-inhibited

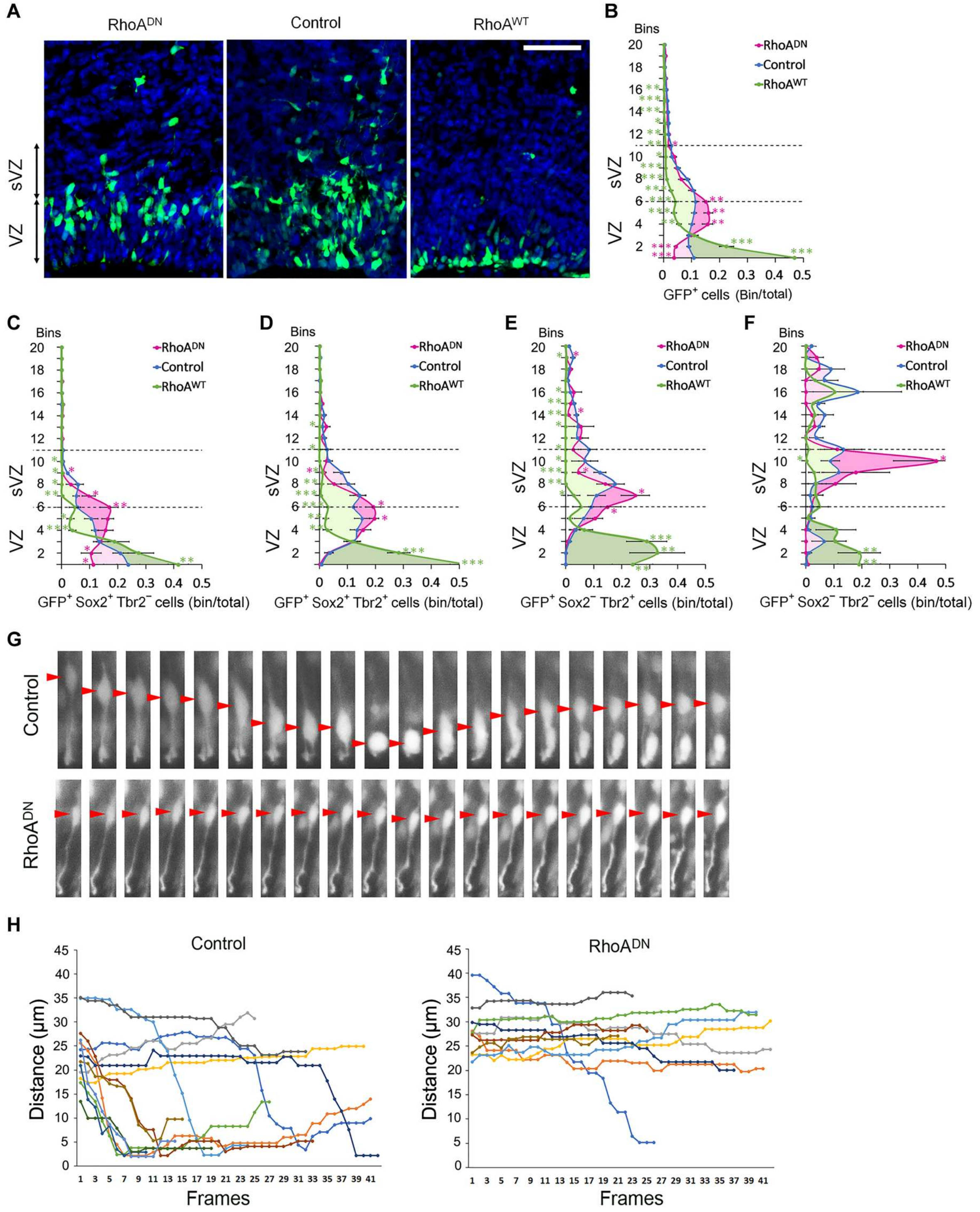

Fig. 2. RhoA regulates the position of RGCs and BPs. (**A**) Coronal sections of E16.5 mice cerebral cortices electroporated with either control, dominant-negative (RhoA^{DN}), or wild-type RhoA (RhoA^{WT}) expression vectors at E15.5, coelectroporated with NLS-GFP, and stained for 4′,6-diamidino-2-phenylindole (DAPI). (**B** to **F**) Graphics of cerebral walls from electroporated brains, corresponding to the VZ to the upper part of the IZ, and subdivided into 20 bins. The graphics indicate the proportion of cells in each bin of (B) GFP$^+$ cells or (C) GFP$^+$Sox2$^+$ Tbr2$^-$ (RGCs), (D) GFP$^+$Sox2$^+$Tbr2$^+$ (Committed BPs), (E) GFP$^+$Sox2$^-$Tbr2$^+$ (BPs), and (F) GFP$^+$Sox2$^-$Tbr2$^-$ (neurons) for control, RhoA^{DN}, or RhoA^{WT} expression vectors electroporated at E15.5 and observed at E16.5. (B) Control n = 21 of 15 IUE (n = 21//15); RhoA^{WT} n = 14//12; RhoA^{DN} n = 20//14; (C to F) Control n = 9//5; RhoA^{WT} n = 7//6; RhoA^{DN} n = 6//6. Error bars, SEM. ***P < 0.001, **P < 0.01, *P < 0.05. Scale bar, 50 μm. (**G** and **H**) Time-lapse analysis of RGCs in the VZ. Frames are every 20 min. Brains were electroporated at E15.5 and processed for videomicroscopy 14 hours later. (G) Representative pictures of the nuclear movement (red arrowheads) followed by control and RhoA-inhibited GFP$^+$ RGCs in the VZ and with a visible apical process attached to the apical surface. (H) Representative nuclear tracks of the paths followed by control and RhoA-inhibited GFP$^+$ RGCs in the VZ with a visible attached apical process. Distance from the ventricular surface of cell centroids in successive frames (circles) is linked by lines.

neurons migrated out of the sVZ (Figs. 1E and 2F and fig. S1L). RhoA GoF resulted in an opposite phenotype with the cell bodies of all four types of cells accumulating at the apical side of the VZ (Figs. 1E and 2, A to F).

There were no overt signs of adherens junction (AJ) weakening after the expression of $RhoA^{DN}$ at E15.5 and examined 1 day later, although AJs were destabilized and disorganized 2 days after surgery (fig. S1, M to O). *RhoA* down-regulation using specific siRNAs mimicked the effect of $RhoA^{DN}$, while *RhoB* knockdown had no noticeable effect on AJs and tissue organization either 1 or 2 days after surgery, which is in agreement with the absence of such phenotype in the *RhoB* knockout mice (*29*, *30*). Overexpression of $RhoA^{WT}$ did not disturb the integrity of AJs 24 hours after surgery (fig. S1, N and O). While consistent with a function of RhoA in AJ stability during mid- to late neurogenesis, these data suggest that the effect on cell positioning measured in this study are observed before any notable AJ disturbance. Because defects in migration could also be due to disturbed radial glia fibers, we performed a Nestin immunolabeling. When observed 1 day after IUE, inhibition or activation of RhoA did not affect the radial glia fibers (fig. S1P).

These results suggest that RhoA activity regulates the position of RGC bodies in the VZ, with a function in the nuclear migration toward the apical surface during the IKNM. They also suggest a function for the migration of committed BPs from the VZ to the sVZ and of neurons out of the sVZ.

The MKL/SRF complex regulates neurogenesis downstream of RhoA

RhoA regulates gene expression through the activation of the SRF transcription factor and its transcription cofactors MKL1 and MKL2 (*38*). In an in vivo luciferase assay on cells electroporated at E15.5 and collected 1 day later, the inhibition of RhoA or a short hairpin RNA (shRNA) targeting both *Mkl1* and *Mkl2* (shMKL1/2) substantially reduced luciferase activity from an SRF-RE reporter plasmid, while RhoA GoF increased its activity (Fig. 3A and fig. S2A). These results suggest that the MKL/SRF pathway is active at E15.5 to E16.5 and might be involved in the observed phenotypes induced by the modulation of RhoA activity.

We first performed epistasis experiments. Mild overexpression of either MKL1 or MKL2 partially restored the differentiation disrupted by the inhibition of RhoA reaching cell-type proportions similar to the control situation (Fig. 3B and fig. S2C). A mild expression of a constitutively activating SRF [SRF fused to a strong transactivation domain from p65NFkappaB (SRF-TAD)] was, however, still too strong when trying to correct the effect of the inhibition of RhoA and led to cell proportions similar to RhoA GoF. The knockdown of *Mkl1* and *Mkl2* partially rescued the differentiation phenotype induced by RhoA GoF (Fig. 3B and fig. S2C). As a control, other shRNAs against MKL1 or MKL2 had similar effects (fig. S2, A to C). The mild expression of a constitutively repressing SRF [SRF fused to the strong repressor domain of Engrailed (SRF-EnRD)] comparably rescued the phenotype induced by an excess of RhoA signal (Fig. 3B and fig. S2C). These data suggest that both MKL1 and MKL2, along with SRF, are involved in cell fate choice controlled by RhoA. As a support to this conclusion, the down- or up-regulation of MKL1, MKL2, and SRF affected neurogenesis similarly to that observed with the manipulation of RhoA activity. shMKL1/2 or the expression of SRF-EnRD decreased the percentage of $GFP^{+}Sox2^{+}$ cells, while GoF for either MKL1, MKL2, or both or the expression of SRF-TAD increased it (fig. S3A).

In line with those analyses, expression of shMKL1/2 or SRF-EnRD reduced the proportion of newly born committed BPs while increasing the amount of BPs and neurons (Fig. 3, C and D, and fig. S2, D and E). Oppositely, MKL2 GoF resulted in an increased percentage of RGCs and committed BPs and a decreased proportion of differentiated BPs and neurons (Fig. 3, C and D). When the overexpression of MKL1 or SRF-TAD was too high, it drastically reduced the presence of Tbr2-expressing cells and consequently increased the percentage of RGCs and neuronal populations (fig. S3B). However, overexpressing a lower dose of MKL1 or a moderate expression of SRF-TAD had an equivalent phenotype as RhoA GoF (Fig. 3, C and D).

As observed with RhoA, cell cycle exit was increased by the knockdown of *Mkl1* and *Mkl2* but decreased by the overexpression of MKL2 or a low dose of MKL1 (Fig. 3E and fig. S2F). In epistasis experiments, MKL1 or MKL2 GoF rescued the effect of RhoA inhibition on the cell cycle exit, while the down-regulation of *Mkl1* and *Mkl2* significantly restored the level of cell cycle exit disrupted by RhoA GoF (Fig. 3E and fig. S2F).

The MKL/SRF complex regulates the position of RGCs and BPs downstream of RhoA

Epistasis experiments showed that SRF-TAD and MKL1, but not MKL2, significantly restored cell positioning disrupted by the inhibition of RhoA (Fig. 4A and figs. S2C and S4). On the other hand, the knockdown of *Mkl1* and *Mkl2*, or of *Mkl1* but not of *Mkl2* alone, or the expression of SRF-EnRD rescued the cell positional defect resulting from RhoA GoF (Fig. 4B and figs. S2C and S4).

In support of the above rescue experiments, manipulating the MKL/SRF pathway phenocopies the results observed when RhoA is affected. Electroporation of either shMKL1/2, shMKL1, or SRF-EnRD, but not shMKL2, induced an increased accumulation of GFP^{+} cells at a zone including the basal side of the VZ and the apical side of the sVZ, a phenotype similar to RhoA inhibition (Figs. 3C and 4C and fig. S3, C to J). Overexpression of MKL1 and MKL2 together, MKL1, or SRF-TAD, but not MKL2 alone, induced the accumulation of GFP^{+} cells at the apical side of the VZ and depletion of cells in the sVZ, similar to the phenotype resulting from RhoA GoF (Figs. 3C and 4C and fig. S3, C to J). Although a milder MKL1 expression had an effect on differentiation, it did not modify cell positioning (Fig. 3, C and D, and fig. S3, K and L), suggesting that cell position and differentiation are sensitive to MKL/SRF dosage. It is likely that high or low levels of MKL/SRF regulate the expression of different sets of genes. Quite unexpectedly, high expression of MKL2 did not recapitulate the apical localization induced by MKL1, SRF-TAD, or RhoA GoF, although it did change cell fate. The change in cell distribution was not extensive but was closer to what we observed when RhoA was inhibited. MKL1 and MKL2 have both common and specific target genes. We believe that MKL2 is not regulating genes involved in cell positioning in the developing neocortex, and it is possible that the overexpression of MKL2 competes with the endogenous MKL1 for its interaction with SRF, inhibiting MKL1 function on cell positioning.

We also investigated the function of the RhoA/MKL/SRF pathway 2.5 days earlier at E13 during early neurogenesis. Although

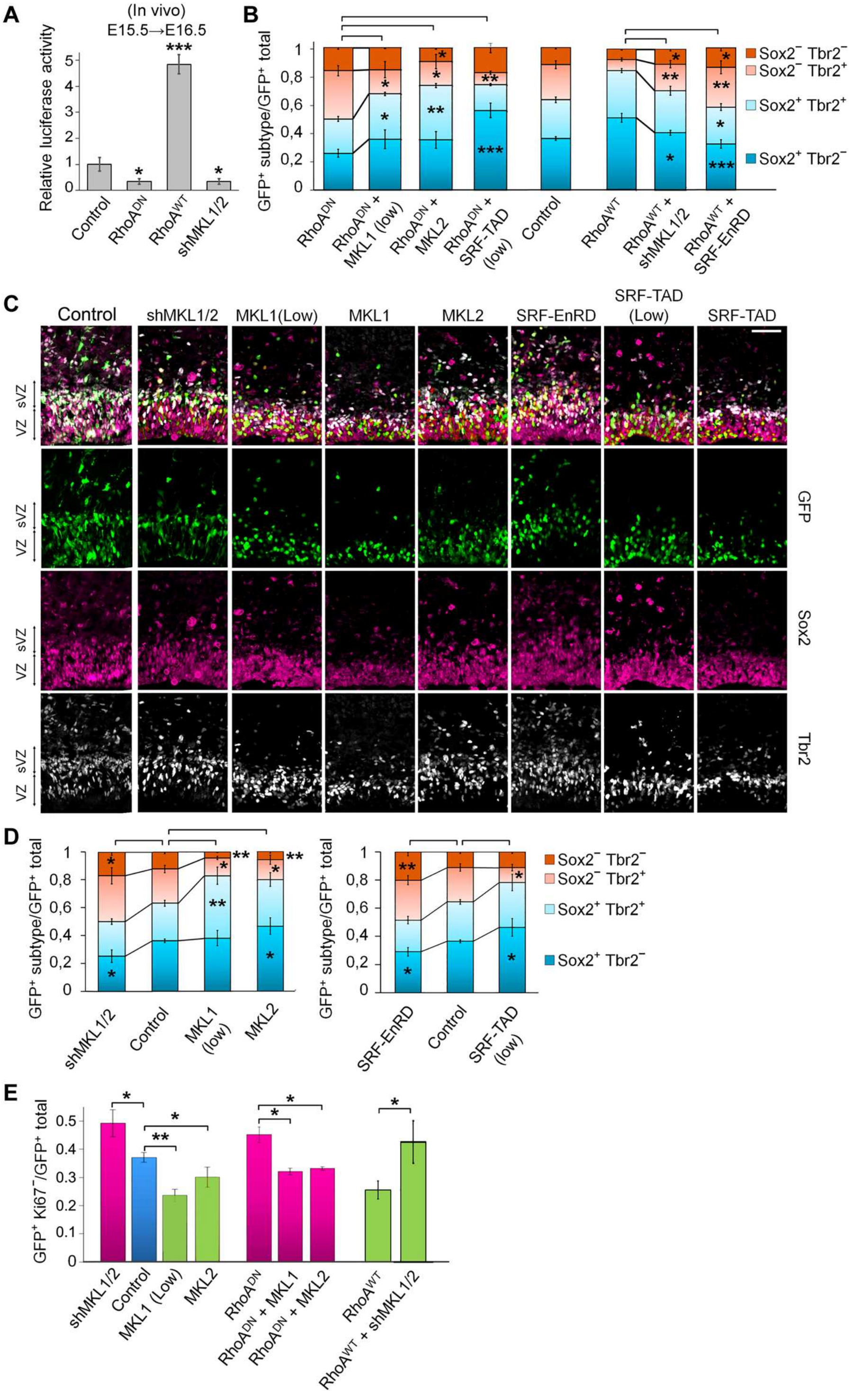

Fig. 3. The MKL/SRF complex regulates neurogenesis downstream of RhoA. (**A**) Luciferase assays were performed on lysates from E16.5 cortices electroporated 1 day earlier with control, $RhoA^{DN}$, $RhoA^{WT}$, or shMKL1/2 expression vectors together with a pGL4-promoter vector containing an SRF-RE that drives transcription of the firefly luciferase reporter gene and a pRL *Renilla* luciferase internal control. Normalized values are reported as the mean fold expression of luciferase activity ± SD from three independent IUE. Relative Luciferase activity in arbitrary units with control set to 1. (**B** to **E**) Brains were in utero electroporated at E15.5 with the indicated plasmids along with NLS-GFP and stained at E16.5 for the indicated markers. (B and D) Quantification of the $GFP^+Sox2^+Tbr2^-$ (RGCs), $GFP^+Sox2^+Tbr2^+$ (committed BPs), GFP^+Sox2^{-} $Tbr2^+$ (BPs), and $GFP^+Sox2^-Tbr2^-$ (neurons) cells. (B) Epistasis experiments are statistically tested against $RhoA^{DN}$ or $RhoA^{WT}$. Controls are shown for comparison. Control n = 9 of 5 IUE (n = 9//5); $RhoA^{WT}$ n = 7//6; $RhoA^{DN}$ n = 8//7; $RhoA^{DN}$ + MKL1(low) n = 4//3; $RhoA^{DN}$ + MKL2 n = 4//3; $RhoA^{DN}$ + SRF-TAD (low) n = 4//3; $RhoA^{WT}$ + shMKL1/2 n = 5//3; $RhoA^{WT}$ + SRF-EnRD n = 8//6. (C) Coronal sections of E16.5 cerebral cortices. (D) Control n = 9//5; shMKL1/2 n = 10//5; MKL1 (low) n = 4//3; MKL2 n = 5//4; SRF-TAD (low) n = 8//3; SRF-EnRD n = 6//3. (E) Quantification of GFP^+Ki67^- cells (exit from cell cycle). Control n = 12//9; $RhoA^{WT}$ n = 9//7; $RhoA^{DN}$ n = 11//6; shMKL1/2 n = 9//4; MKL1 (low) n = 4//3; MKL2 n = 6//5; $RhoA^{WT}$ + shMKL1/2 n = 3//3; $RhoA^{DN}$ + MKL1 n = 4//3; $RhoA^{DN}$ + MKL2 n = 4//3. Error bars, SEM. ***$P < 0.001$, **$P < 0.01$, *$P < 0.05$. Scale bar, 50 μm.

the effect was not as strong as at E15.5, we observed similar effects on cell positioning and neurogenesis when manipulating RhoA or MKL activities (fig. S5). Overall, these data support a common function for RhoA, MKL1, MKL2, and SRF in neurogenesis, while MKL1 and SRF, but not MKL2, are downstream of RhoA in the signaling pathway regulating the positioning of RGCs, BPs, and neurons.

Bcl6 and SRF physically interact and form a complex with MKL1 or 2

To screen for potential neurogenic transcription factors capable of counteracting the differentiation and position phenotypes induced by the modification of RhoA/MKL/SRF activity, we used a sequence-based bioinformatics tool for prediction of transcriptional regulation interactors (*55*). This led us to focus on Bcl6. *Bcl6* is

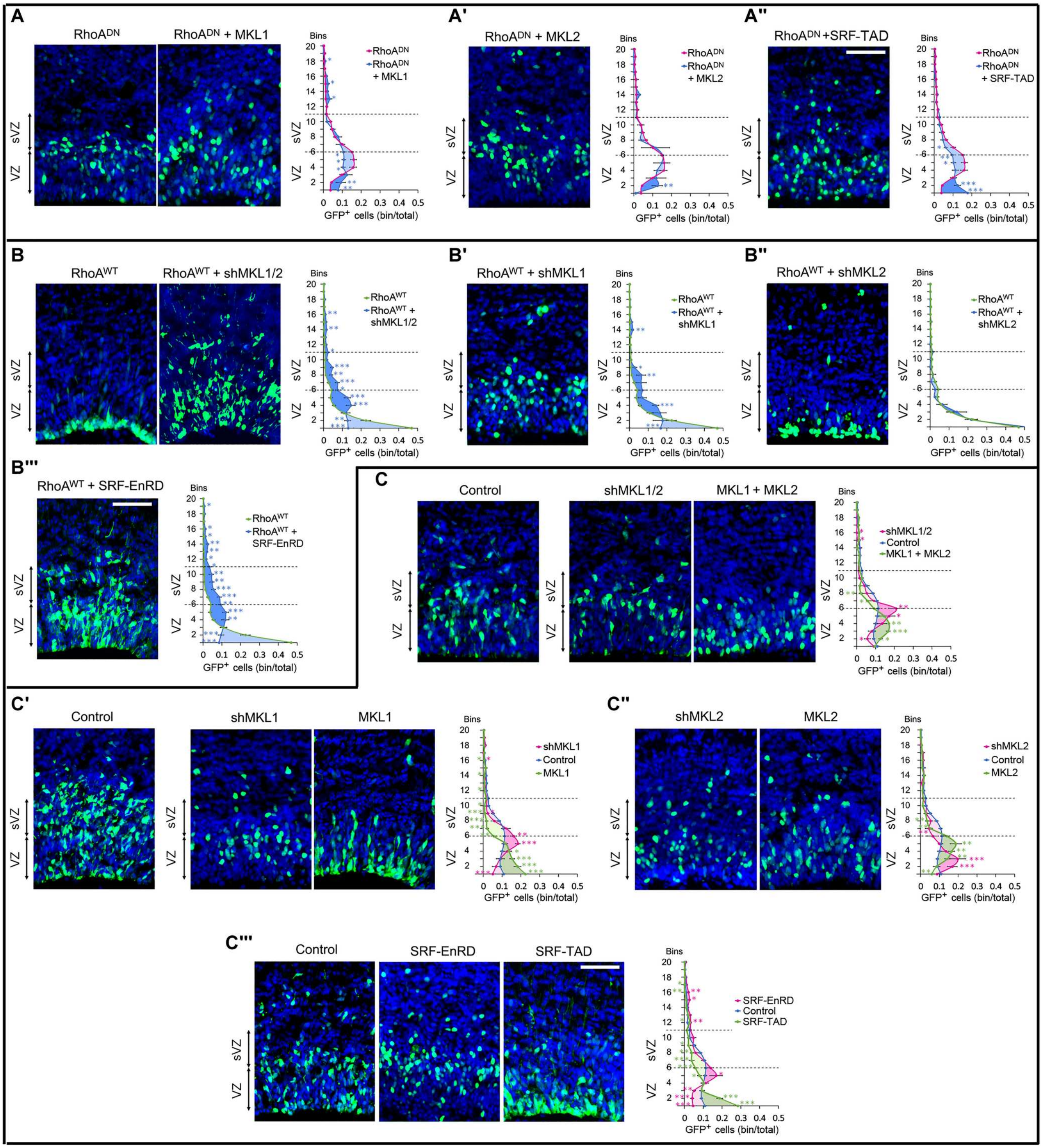

Fig. 4. The MKL/SRF complex regulates the position of RGCs and BPs downstream of RhoA. E16.5 Coronal sections of mice cerebral cortices electroporated at E15.5 with the indicated plasmids along with NLS-GFP expression vector and stained with DAPI. The associated graphics show the radial distribution of GFP$^+$ cells. (**A**, **A**′, and **A**″) MKL1 and SRF-TAD but not MKL2 rescued the positioning defect induced by RhoA^{DN}; RhoA^{DN} *n* = 20 of 14 IUE (*n* = 20//14); RhoA^{DN} + MKL1 *n* = 5//3; RhoA^{DN} + MKL2 *n* = 4//3; RhoA^{DN} + SRF-TAD *n* = 6//3. (**B**, **B**′, **B**″, and **B**‴) shMKL1/2, shMKL1, and SRF-EnRD but not shMKL2 rescued the positioning defect induced by RhoA^{WT}; RhoA^{WT} *n* = 14//12; RhoA^{WT} + shMKL1/2 *n* = 8//3; RhoA^{WT} + shMKL1 *n* = 15//5; RhoA^{WT} + shMKL2 *n* = 5//3; RhoA^{WT} + SRF-EnRD *n* = 12//6; (**C**, **C**′, **C**″, and **C**‴) Inhibition or GoF of MKL1 and SRF but not MKL2 induced similar positional phenotypes as inhibition or GoF of RhoA. Control *n* = 21//15; shMKL1/2 *n* = 10//5; MKL1 + MKL2 *n* = 5//3; shMKL1 *n* = 9//3; MKL1 *n* = 12//6; shMKL2 *n* = 4//3; MKL2 *n* = 6//6; SRF-EnRD *n* = 10//6; SRF-TAD *n* = 9//4. Error bars, SEM. ****P* < 0.001, ***P* < 0.01, **P* < 0.05. Scale bar, 50 μm.

encoding a zinc finger transcriptional repressor. This protein was previously found to act on neurogenesis in the developing mammalian cerebral cortex (*45*, *46*, *56*). We first investigated protein interactions using trimolecular fluorescence complementation (TriFC) assay where the direct interaction of two tagged proteins induces fluorescence emission (*57*). Positive controls showed that MKL1 and MKL2 successfully interact with SRF, as observed by the presence of green fluorescence in the nuclei (Fig. 5, A and B). MKL1 is known to dimerize through its LZ domain (*58*). We therefore used a mutant MKL1 lacking the LZ domain [MKL1(ΔLZ)] as a negative control. MKL1(ΔLZ) interaction to MKL1 was strongly reduced compared to its wild type version. A very distinct nuclear interaction was observed between Bcl6 and SRF, while no fluorescence was detected when MKL1 or MKL2 were cotransfected with Bcl6 (Fig. 5, A and B).

In contrast to the TriFC approach, coimmunoprecipitation allows the detection of multiple proteins in the same complex. Coimmunoprecipitation experiments in human embryonic kidney (HEK) 293T cells confirmed a direct interaction between Bcl6 and SRF but not MKL1 (Fig. 5, C to E). When the three proteins were overexpressed together, Bcl6 did not prevent MKL1 or MKL2 interaction with SRF but was coimmunoprecipitated with the MKL/SRF complex when MKL1 or MKL2 was pulled down (Fig. 5E and fig. S6). These results show that Bcl6 most likely interacts directly with SRF, whereas the interaction with MKL1 or MKL2 is indirect and requires them to be in complex with SRF. Unfortunately, the endogenous complex formation could not be tested. Several SRF and Bcl6 antibodies were used and were unable to immunoprecipitate the endogenous proteins.

Bcl6 and MKL/SRF mutually repress their transcriptional activity in vitro and in vivo

We performed luciferase assays using an SRF-RE vector in vitro in HEK293T cells or in vivo in E15.5 embryonic cortices. In cells, the amount of signal induced by MKL1 decreased when Bcl6–hemagglutinin (HA) was coexpressed, while the decrease was not significant on the signal induced by MKL2 (Fig. 5F). In vivo, the luciferase signal was tested 23 hours after surgery. Overexpression of Bcl6 slightly but significantly reduced the endogenous activity on the SRF-RE sensor, while the knockdown of *Bcl6* increased it (Fig. 5G).

To test whether there was a reciprocal effect of MKL/SRF on Bcl6, we constructed a luciferase sensor [Bcl6(BS)-Luc] containing the consensus Bcl6 binding site sequence in front of the promoter as used in (*59*–*61*). As expected from a transcriptional repressor, Bcl6 inhibited the luciferase expression level in HEK293T cells. We then cotransfected either $RhoA^{WT}$, $RhoA^{DN}$, or MKL1 with Bcl6 to determine whether they had any synergistic or antagonistic effect. Our results showed that MKL1 or $RhoA^{WT}$ coexpression inhibited the repression activity of Bcl6. Inhibition of RhoA via $RhoA^{DN}$ had no significant effect (Fig. 5H). We then expressed the Bcl6(BS)-Luc sensor in vivo and found that MKL1 and $RhoA^{WT}$ prevented the repressor activity of the endogenous Bcl6 on the sensor, while inhibition of RhoA enhanced it (Fig. 5I). Overall, these results suggest that Bcl6 and the RhoA downstream effectors MKL/SRF mutually repress their transcriptional activity in vitro and in vivo.

Bcl6 and SRF interaction prevents each other's homodimerization

We next investigated the mechanism by which this interaction inhibits MKL/SRF and Bcl6 activities. Some transcription cofactors such as MKLs shuttle between the cytoplasm and the nucleus (*62*). We examined Bcl6 and MKL subcellular localization when co-overexpressed in HeLa cells (fig. S7). Proteins were categorized as essentially cytoplasmic (at least 60% of the signal in the cytoplasm), intermediate (the repartition of the fluorescence in the nucleus and the cytoplasm was between 40 and 60%), or essentially nuclear (at least 60% of the signal in the nucleus). Overexpressing Bcl6 partially displaced both MKL1 and MKL2 toward the cytoplasm (fig. S7). On the other hand, RhoA activation or inhibition, or co-overexpression of MKL1 or MKL2, did not modify Bcl6 predominant nuclear location.

Both SRF and Bcl6 homodimerize to fully accomplish their function (*63*, *64*). Preventing their homodimerization would therefore be an inhibitory mechanism. We found that Bcl6 overexpression reduced SRF, but not MKL1, homodimerization, while it slightly but nonsignificantly affected MKL1 and SRF heterodimer formation. On the other hand, SRF overexpression strongly decreased Bcl6 dimer formation (Fig. 5, E, J, and K). These data suggest that SRF and Bcl6 interaction inhibits each other's homodimer formation.

We further characterized SRF and Bcl6 heteromer formation and investigated specific domain interactions. Using the TriFC approach, we tested the ability of the known domains of SRF to bind to the full-length Bcl6 and, vice versa, we tested whether the known Bcl6 domains interact with the full-length SRF. Our results are schematically summarized in Fig. 6. Only the truncated SRF proteins that contain the amino acids 1 to 168 were able to associate with Bcl6. This region contains the SRF repressor domain (*65*, *66*). As a control, we confirmed that MKL1 binds to the DNA binding domain of SRF (*58*). The TAD domain of SRF did not interact with Bcl6 or MKL1. We also found that the Bcl6-truncated proteins containing either the BTB domain or a central region of Bcl6 that overlap with both the repressor domain 2 and the PEST domains of Bcl6 (*67*) were able to interact with SRF, while the Zn-Finger domain of Bcl6 was not supporting an interaction with SRF. It is not unusual to observe two domains within a protein involved in interaction with another protein (*68*, *69*).

Bcl6 opposes the functions of RhoA and MKL/SRF on neocortical cell differentiation and position in vivo

So far, our data indicate that the RhoA/MKL/SRF pathway and Bcl6 have opposite functions and suggest that they regulate each other in vivo. To support a functional interaction between the RhoA/MKL/SRF pathway and Bcl6, we performed epistasis experiments in vivo (Fig. 7 and figs. S8 and S9). The knockdown of *Bcl6* rescued the differentiation, the level of cell cycle exit, and the position phenotypes induced by RhoA, MKL, or SRF inhibition. The use of another shRNA targeting a different sequence of Bcl6 induced a similar rescue (figs. S10). Moreover, Bcl6 overexpression partially restored cell position, cell cycle exit, and differentiation disrupted by RhoA, MKL, or SRF GoF (Fig. 7 and figs. S8 and S9).

In agreement with the above epistasis experiments, the knockdown of *Bcl6* alone reduced the proportion of differentiated cells and decreased cell cycle exit, while it did not affect the mitotic index when electroporated at E15.5 and observed 1 day later

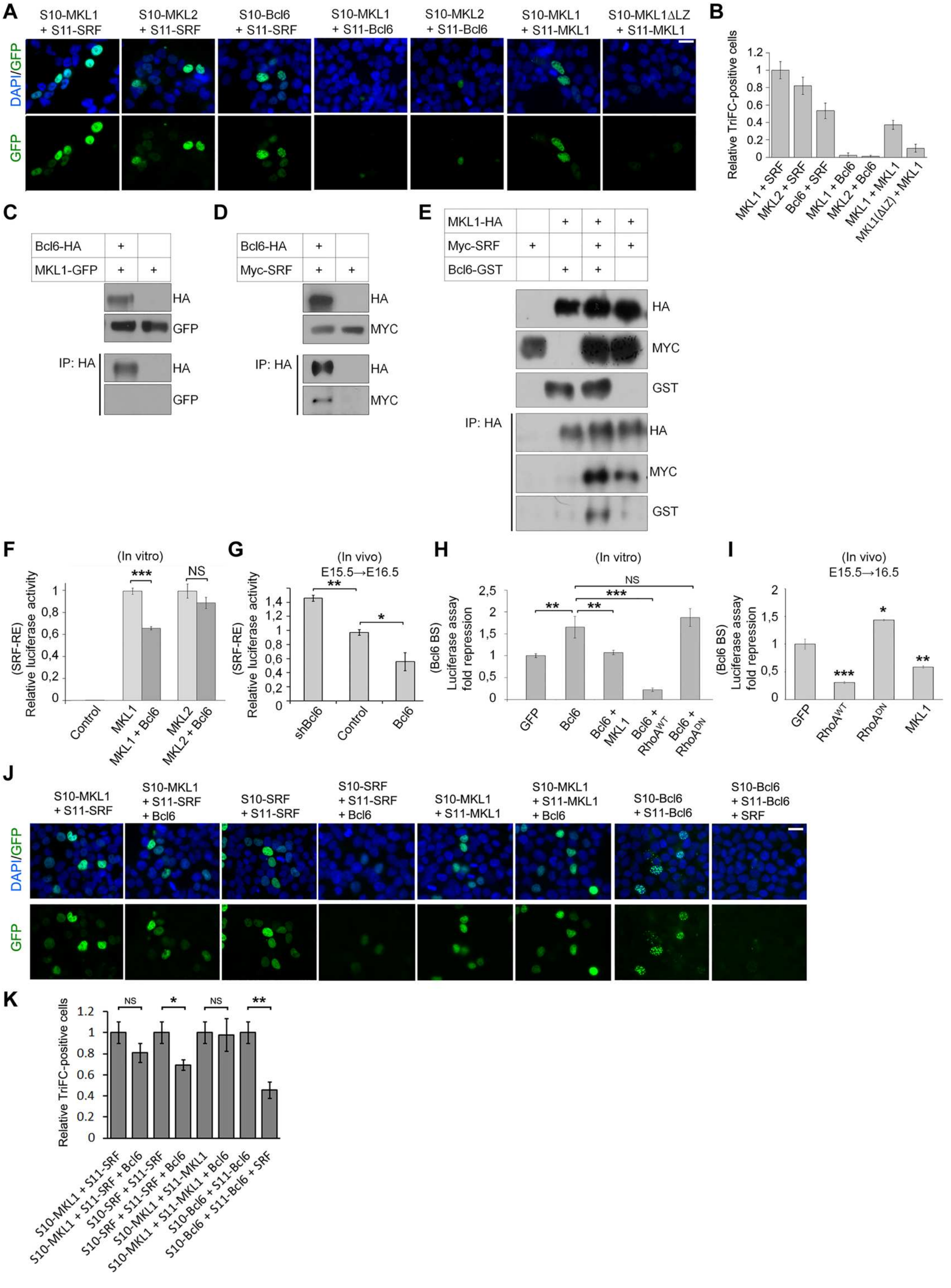

Fig. 5. Bcl6 and MKL/SRF mutually repress their transcriptional activity in vitro and in vivo through the formation of a tripartite complex that depends on Bcl6 and SRF direct physical interaction, which prevents each other's homodimerization. (**A**, **B**, **J**, and **K**) TriFC assay. Epifluorescence microscopic images of HEK293T cells 8 hours after transfection for the coexpression of the GFP (S1 to S9) fragment and the indicated proteins fused with either S10 or S11 GFP fragments and untagged proteins. Cells were stained with DAPI. The graphics indicate the relative GFP complementation with the condition "SRF + MKL1" set at 1 ± SD from three independent experiments. (**C** to **E**) Coimmunoprecipitation assays were performed using tagged Bcl6, SRF, and MKL1 expressed in HEK293T. Twenty-four hours after transfection, cell lysates were analyzed directly or after pull-down with an HA antibody. Samples were immunoblotted with HA, Myc, or glutathione *S*-transferase (GST) antibodies. $n \geq 3$. (**F** to **I**) Luciferase assays performed with either control, $RhoA^{WT}$, $RhoA^{DN}$, MKL1, MKL2, shBcl6, or Bcl6 expression vectors together with a pRL *Renilla* luciferase internal control and (F and G) a pGL4-promoter vector containing SRF-RE (results are expressed as relative fold-change of luciferase activity) or (H and I) a pTA vector containing a Bcl6(BS) in front of a cytomegalovirus (CMV) promoter that drives the transcription of the firefly *luciferase* reporter gene (normalized values are reported as the mean fold repression of luciferase activity). The luciferase assays were performed (F and H) on HEK293T lysates 24 hours after transfection or (G and I) on lysates from E16.5 cortices electroporated 1 day earlier. Relative luciferase activity in arbitrary units ± SD from three independent experiments. Error bars, SEM. ***$P < 0.001$, **$P < 0.01$, *$P < 0.05$, NS, not significant. Scale bar, 50 μm.

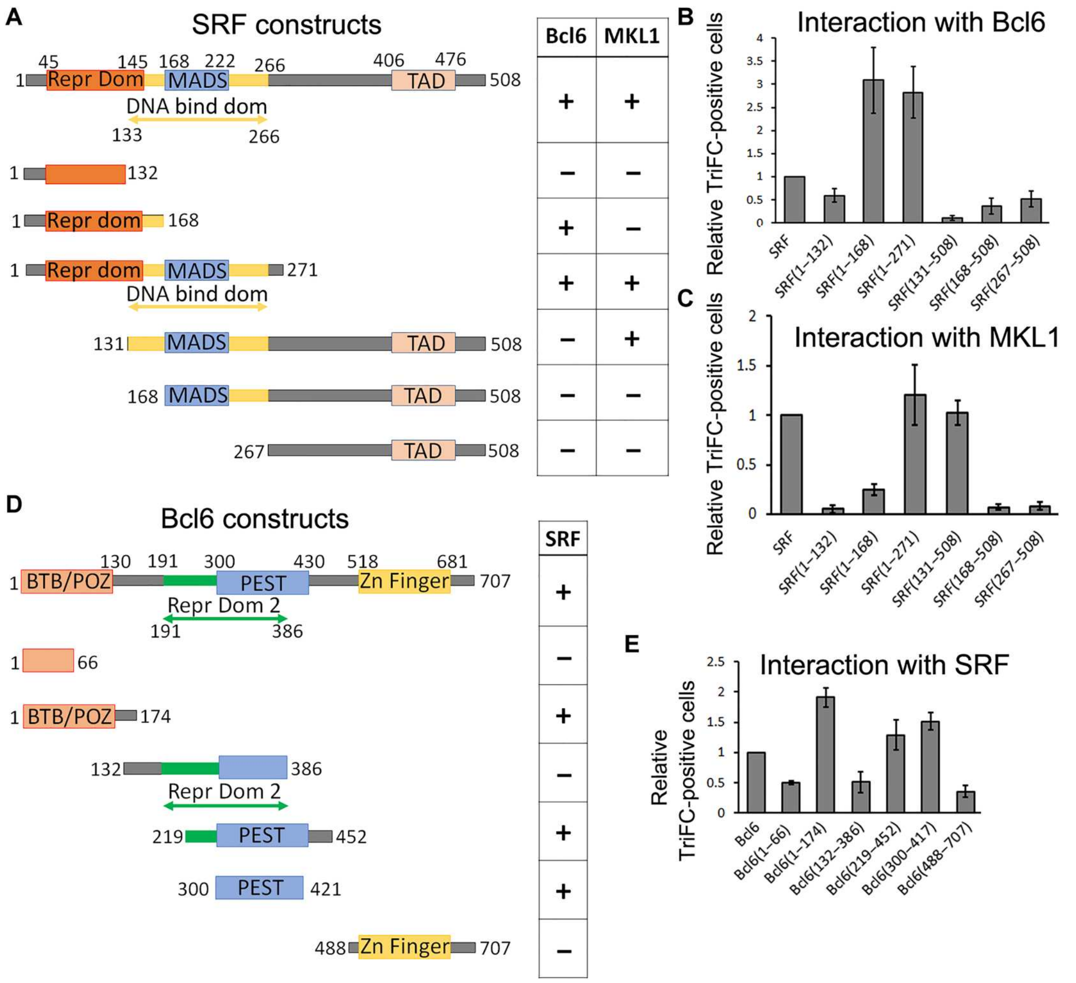

Fig. 6. Identification of Bcl6 and SRF domains involved in their interaction. (**A** and **D**) Schematic representation of the different domain constructions. (**B**, **C**, and **E**) TriFC assay. The graphics indicate the comparison of fluorescence from GFP complementation with the conditions "SRF + Bcl6" in (B), "SRF + MKL1" in (C), and "Bcl6 + SRF" in (E) set at 1. *n* = 8 independent experiments. Error bars, SEM.

(Fig. 8). Bcl6 GoF on its own increased the production of differentiated cells and cell cycle exit but had almost no effect on cell positioning.

Bcl6 and SRF physical interaction is necessary for RhoA/MKL/SRF pathway and Bcl6 mutual inhibition in vitro and in vivo

To validate the significance of Bcl6/SRF interaction in vivo, we introduced point mutations in the two SRF-binding domains of Bcl6 (Fig. 9A). We carefully selected mutations that weakened the interaction in a TriFC assay while ensuring that they did not diminish Bcl6 activity in a luciferase assay using a Bcl6 sensor. Two mutations, one in the BTB and one in the PEST domains of Bcl6, significantly reduced its interaction with SRF (Fig. 9B). The mutated Bcl6 did not lose but rather showed an increase in transcriptional repressive activity (Fig. 9C). We then showed that this Bcl6 mutant lost its ability to inhibit MKL1/SRF transcriptional activity in vitro and in vivo (Fig. 9, D and E). Furthermore, this version of Bcl6 unable to interact with SRF could not rescue the positional phenotypes induced by RhoA GoF (Fig. 9, F and G, and fig. S11). Bcl6-SRF interaction is therefore essential for Bcl6 to counteract RhoA control of cell positioning. On the other hand, the Bcl6 mutant form was as efficient as Bcl6 wild type to oppose the defect in cell cycle exit resulting from an excess of RhoA (Figs. 9H and 7F for Bcl6 wild type). Concerning neurogenesis, the rescue was only partial, with an effect on $Sox2^{+}$ cells and neurons (Fig. 9, I and K), while the proportion of $Tbr2^{+}$ cells was not restored (Fig. 9J). This indicates that Bcl6 interaction with SRF is not necessary to oppose RhoA effect on cell cycle exit and direct neurogenesis but is required to regulate $Tbr2^{+}$ BP production (indirect neurogenesis). In agreement with these results, overexpression of the Bcl6 mutant had similar effects to the wild-type Bcl6 on cell cycle exit (Figs. 9N and 8C for Bcl6 wild type) and direct neurogenesis but had an opposite influence on $Tbr2^{+}$ cells (Fig. 9, O to Q, and Fig. 8B). While RhoA GoF could rescue the impact of Bcl6 overexpression on cell cycle exit (Fig. 9H), $Sox2^{+}$ cells (Fig. 9I), $Tbr2^{+}$ cells (Fig. 9J), neuronal production, and the overall proportion of the four cell types studied (Fig. 9K), it could not restore these changes resulting from the overexpression of the interaction-defective Bcl6 form (Fig. 9, H

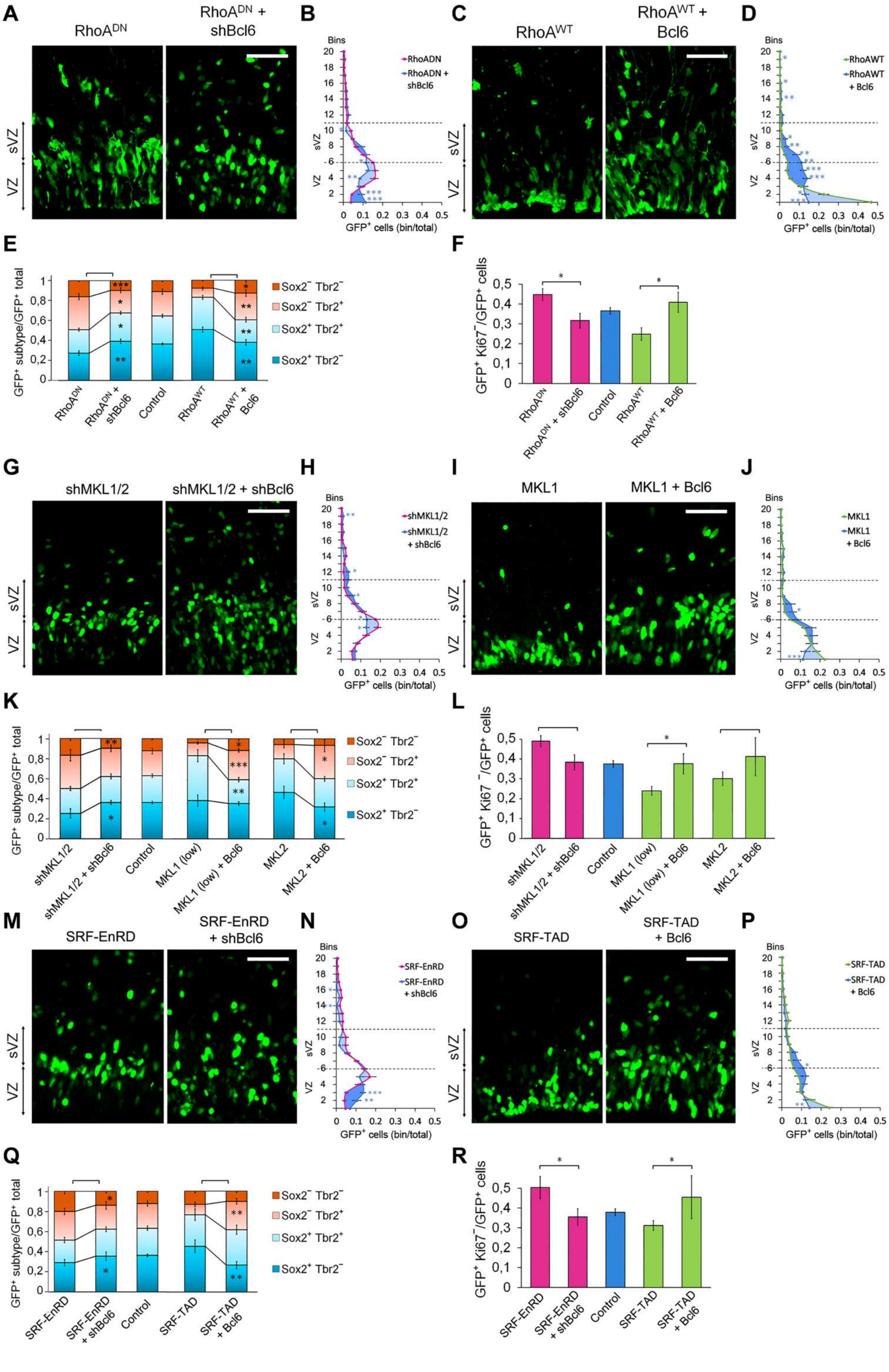

Fig. 7. Bcl6 activity counteracts the effect of RhoA on neurogenesis and cell position in vivo. (**A**, **C**, **G**, **I**, **M**, and **O**) Coronal sections of E16.5 cerebral cortices in utero electroporated at E15.5 with the indicated plasmids along with NLS-GFP. (**B**, **D**, **H**, **J**, **N**, and **P**) The graphics indicate the proportion of cells in each bin of GFP$^+$ cells for the indicated expression vectors electroporated at E15.5 and observed at E16.5. (B) RhoA^{DN} n = 20 of 14 IUE (n = 20//14); RhoA^{DN} + shBcl6 n = 10//8; (D) RhoA^{WT} n = 14//12; RhoA^{WT} + Bcl6 n = 7//5; (H) shMKL1/2 n = 10//5; shMKL1/2 + shBcl6 n = 11//4; (J) MKL1 n = 12//7; MKL1 + Bcl6 n = 4//3; (N) SRF-EnRD n = 10//6; SRF-EnRD + shBcl6 n = 5//3; (P) SRF-TAD n = 9//4; SRF-TAD + Bcl6 n = 7//3. (**E**, **F**, **K**, **L**, **Q**, and **R**) Quantification of the GFP$^+$Sox2$^+$Tbr2$^-$, GFP$^+$Sox2$^+$Tbr2$^+$, GFP$^+$Sox2$^-$Tbr2$^+$, GFP$^+$Sox2$^-$Tbr2$^-$, and GFP$^+$ Ki67$^-$ cells. (E) Control n = 9//5; RhoA^{WT} n = 7//6; RhoA^{DN} n = 8//7; RhoA^{DN} + shBcl6 n = 10//4; RhoA^{WT} + Bcl6 n = 9//5; (F) Control n = 12//9; RhoA^{WT} n = 9//7; RhoA^{DN} n = 11//6; RhoA^{DN} + shBcl6 n = 12//6; RhoA^{WT} + Bcl6 n = 9//5; (K) Control n = 9//5; shMKL1/2 n = 7//4; MKL1 (low) n = 4//3; MKL2 n = 5//4; shMKL1/2 + shBcl6 n = 8//3; MKL1 (low) + Bcl6 n = 10//4; MKL2 + Bcl6 n = 3//3; (L) Control n = 12//9; shMKL1/2 n = 6//3; MKL1 (low) n = 4//3; MKL2 n = 6//5; shMKL1/2 + shBcl6 n = 15//5; MKL1 (low) + Bcl6 n = 10//4; MKL2 + Bcl6 n = 3//3; (Q) Control n = 9//5; SRF-TAD n = 8//3; SRF-EnRD n = 6//3; SRF-EnRD + shBcl6 n = 7//3; SRF-TAD+Bcl6 n = 5//3; (R) Control n = 12//9; SRF-TAD n = 6//3; SRF-EnRD n = 7//4; SRF-EnRD + shBcl6 n = 6//3; SRF-TAD +Bcl6 n = 6//3. Error bars, SEM. ***$P < 0.001$, **$P < 0.01$, *$P < 0.05$. Scale bar, 50 μm.

to K). These data suggest that SRF/Bcl6 interaction is necessary for RhoA to regulate neurogenesis through Bcl6.

RhoA-, MKL1-, and SRF-induced lengthening of the G_1 phase of the cell cycle does not influence neurogenesis and is not opposed by Bcl6

The IKNM movement along the apical-basal axis of RGCs nuclei occurs in concert with the cell cycle (*8*). In addition, the length of the cell cycle and, more specifically, the length of the G_1 phase have been shown to influence symmetrical (self-expansion) versus

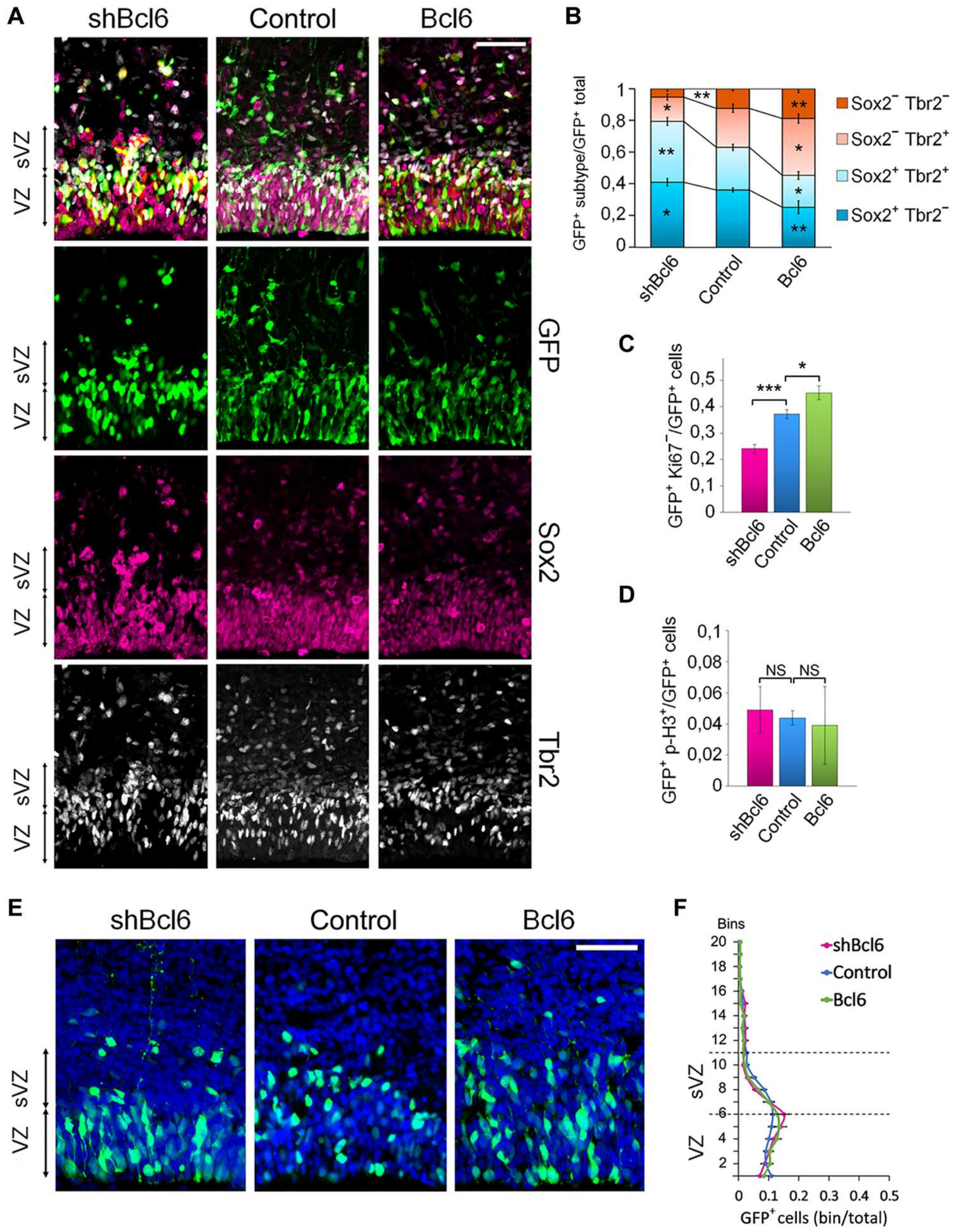

Fig. 8. Bcl6 induces neurogenesis in vivo. E15.5 brains were electroporated with the indicated plasmids along with NLS-GFP and processed at E16.5. (**A** and **E**) Coronal sections stained for the indicated markers. (**B** to **D**) Quantification of the $GFP^+Sox2^+Tbr2^-$, $GFP^+Sox2^+Tbr2^+$, $GFP^+Sox2^-Tbr2^+$, $GFP^+Sox2^-Tbr2^-$, GFP^+Ki67^-, and GFP^+p-$H3^+$ cells. (B) Control *n* = 9 of 5 IUE (*n* = 9//5); shBcl6 *n* = 4//3; Bcl6 *n* = 5//3; (C) Control *n* = 12//9; shBcl6 *n* = 5//5; Bcl6 *n* = 13//7; (D) Control *n* = 7//4; shBcl6 *n* = 3//3; Bcl6 *n* = 12//6. (**F**) The graphics indicate the percentage of cells in each bin of GFP^+ cells from brains electroporated with the indicated expression vectors. Control *n* = 21//15; shBcl6 *n* = 10//7; Bcl6 *n* = 12//6. Error bars, SEM. ***$P < 0.001$, **$P < 0.01$, *$P < 0.05$; NS, not significant. Scale bar, 50 μm.

asymmetrical (neurogenic) division in early NSCs (*70*). On the basis of our findings showing a negative effect of RhoA on neurogenesis and a perturbation of the IKNM of RGCs, we reasoned that RhoA might influence the G_1 phase of the cell cycle.

During an IUE, only RGCs in their S or G_2-M phases receive plasmids (*51*, *52*). Consequently, the first GFP^+ cells to incorporate 5-ethynyl-2-deoxyuridine (EdU) after the surgery are the ones in M phase at the time of electroporation (Fig. 10A). The following experimental procedure allows the measurement of the minimum G_1 length (*51*, *52*). A 30-min EdU pulse was performed 8, 10, 12, 18, and 23 hours after IUE at E15.5 (Fig. 10, B to E). Eight hours after electroporation, none of the GFP^+ cells in the tested conditions were positive for EdU. The first GFP^+ EdU^+ cells were observed at the 10-hour EdU pulse in the RhoA-inhibited condition, at 12 hours in the control brains, and only at 18 hours in the RhoA GoF brains (Fig. 10, B and C). These data indicate that the G_1 phase lasts approximately 12 hours in control RGCs. RhoA inhibition shortened the G_1 phase by 2 hours, while the G_1 phase in RhoA GoF cells was increased by 4 hours.

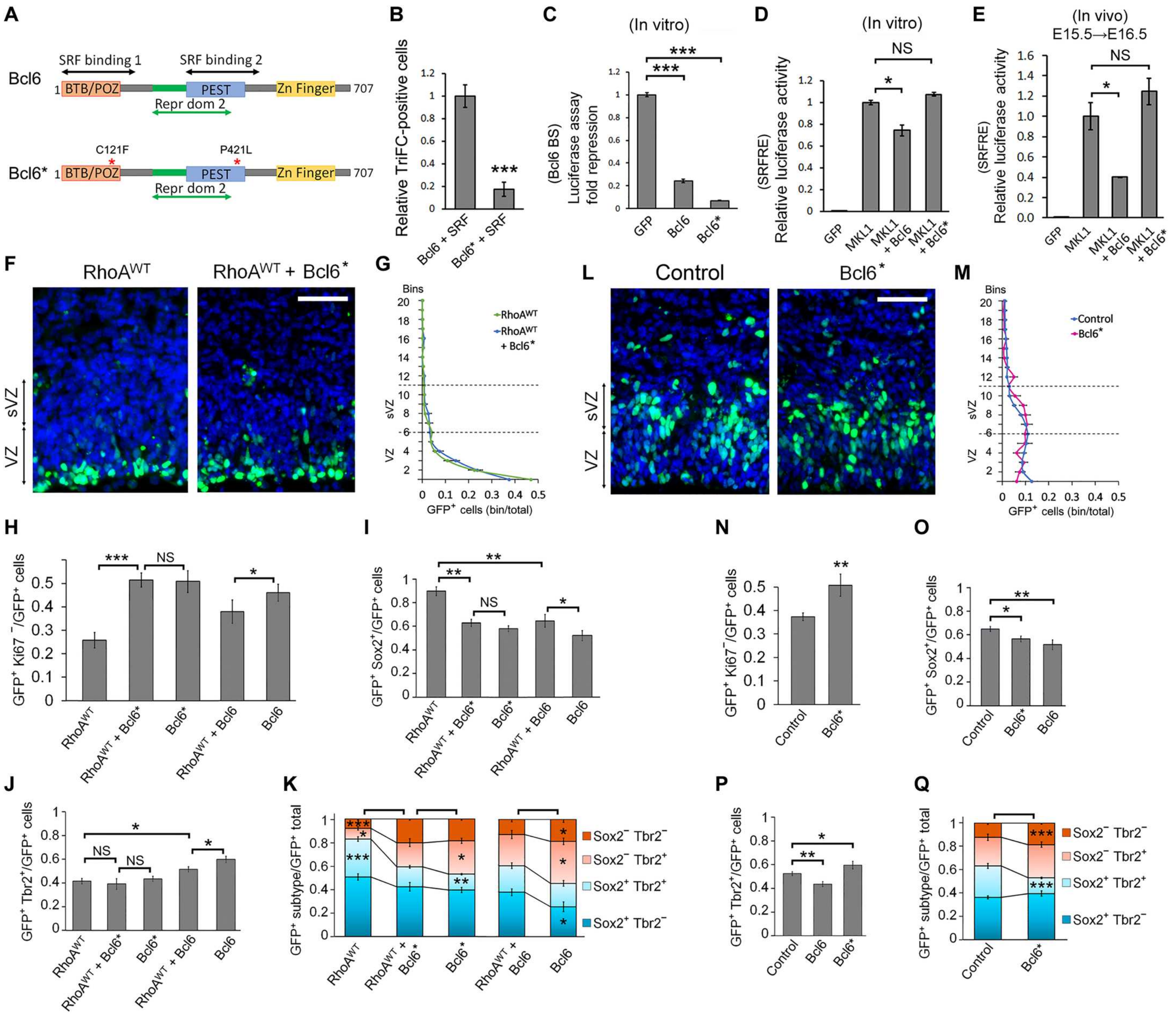

Fig. 9. Bcl6 and SRF physical interaction is necessary for the RhoA/MKL/SRF pathway and Bcl6 mutual inhibition in vitro and in vivo. (**A**) Schematic representation of Bcl6 protein structure and location of mutations in the Bcl6 mutant (Bcl6*). (**B**) TriFC assay. The graphics indicate the percentage of GFP^+ complemented cells with the condition "Bcl6 + SRF" set at 100%. $n = 4$. (**C** to **E**) Luciferase assays performed with either control, MKL1, Bcl6, or Bcl6 bearing the mutations C121F and P421L (Bcl6*) expression vectors together with a pRL *Renilla* luciferase internal control and a pGL4-promoter vector containing (C) a pTA vector containing a Bcl6(BS) in front of a CMV promoter that drives the transcription of the firefly *luciferase* reporter gene (normalized values are reported as the mean fold repression of luciferase activity) or (D and E) as SRF-RE (results are expressed as relative fold-change of luciferase activity in arbitrary units). The luciferase assays were performed (C and D) on HEK293T lysates 24 hours after transfection or (E) on lysates from E16.5 cortices electroporated 1 day earlier. $n = 3$. (**F** and **L**) Coronal sections of E16.5 cerebral cortices in utero electroporated at E15.5 with the indicated plasmids along with NLS-GFP. (**G** and **M**) The graphics indicate the proportion of cells in each bin of GFP^+ cells for the indicated expression vectors electroporated at E15.5 and observed at E16.5. $RhoA^{DN}$ $n = 20$ of 14 IUE ($n = 20//14$); $RhoA^{DN}$ + Bcl6* $n = 6//3$; Control $n = 21//15$; Bcl6* $n = 11//4$. (**H** to **K** and **N** to **Q**) Quantification of the GFP^+Ki67^-; GFP^+Sox2^+; GFP^+Tbr2^+; $GFP^+Sox2^+Tbr2^-$; $GFP^+Sox2^+Tbr2^+$; $GFP^+Sox2^-Tbr2^+$; $GFP^+Sox2^-Tbr2^-$; cells. (H and N) $RhoA^{WT}$ $n = 9//7$; $RhoA^{WT}$ + Bcl6* $n = 4//3$; $RhoA^{WT}$ + Bcl6 $n = 9//5$; Control $n = 12//9$; Bcl6 $n = 13//7$; Bcl6* $n = 9//3$; (I to K and O to Q) $RhoA^{WT}$ $n = 7//6$; $RhoA^{WT}$ + Bcl6* $n = 9//3$; Bcl6* $n = 11//4$; $RhoA^{WT}$ + Bcl6 $n = 9//5$; Bcl6 $n = 5//3$; Control $n = 9//5$. Error bars, SEM; ***$P < 0.001$, **$P < 0.01$, *$P < 0.05$; NS, not significant. Scale bar, 50 μm.

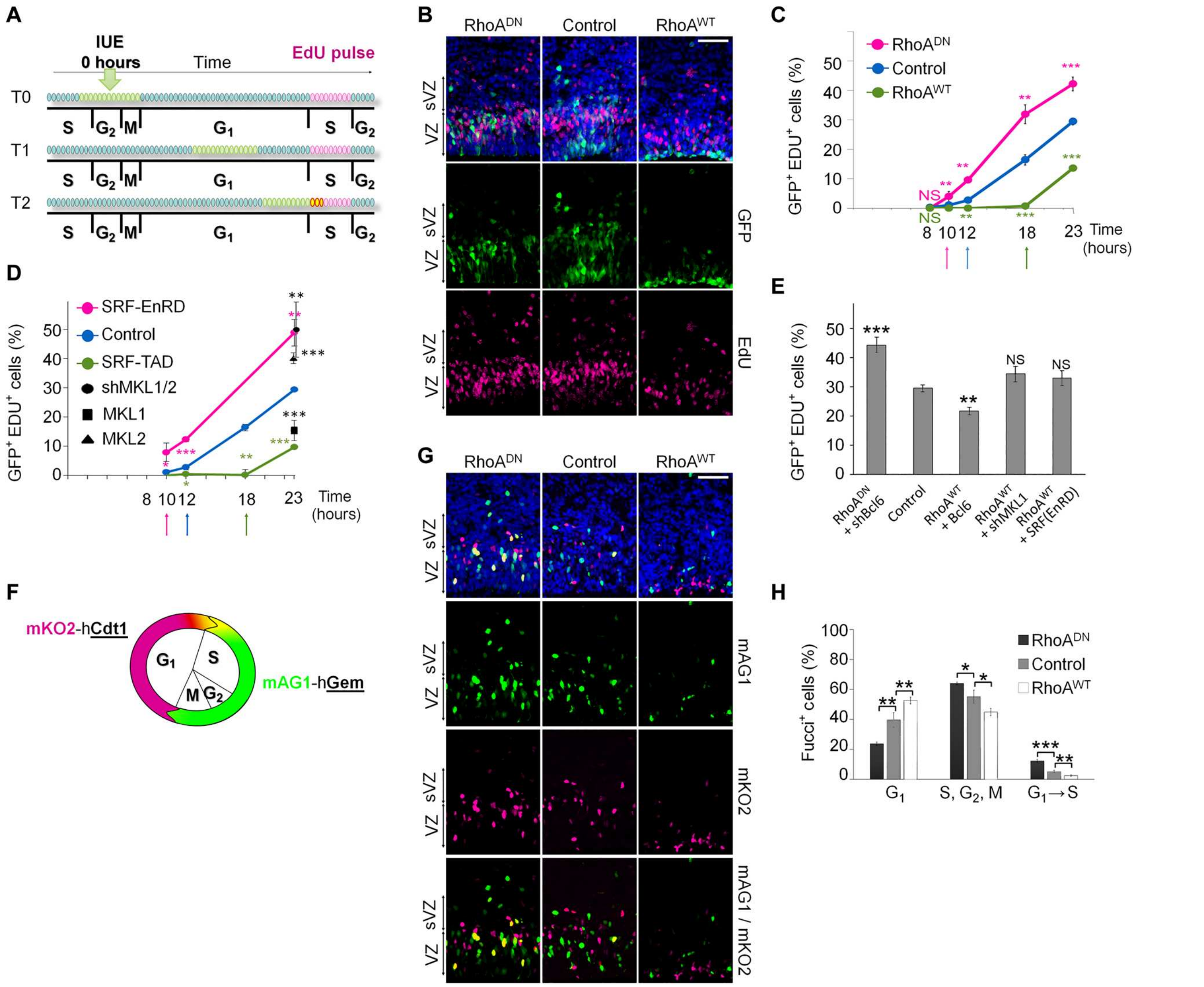

Fig. 10. RhoA, MKL, and SRF increase the length of the G_1 phase of the cell cycle. (**A**) Representative diagram of the G_1 phase length measurement protocol. Electroporation transfects a cohort of cells in late S, G_2-M phases (green circles) at time T0. The GFP^+ cohort progresses through the G_1 phase and observed at different timing (example here, T1 or T2). A 30-min EdU pulse before dissection at T1 or T2 labels S phase cells (red circles). T2 represents the time for electroporated cells to reenter the S phase and observed by the earliest appearance of double staining for GFP and EdU (yellow circles). (**B** to **H**) Brains were in utero electroporated at E15.5. (B) Coronal sections of E16.5 cerebral cortices electroporated with either a control, $RhoA^{DN}$, or $RhoA^{WT}$ expression plasmids at E15.5 along with NLS-GFP and stained for EdU. (C to E) Quantification of GFP^+EdU^+ cells after a single 30-min EdU injection at 8, 10, 12, 18, or 23 hours after surgery. $n \geq 5$ for EdU at 23 hours; $n \geq 3$ for EdU at 8, 10, 12, and 18 hours after surgery. (F) Illustration of the regulation of the expression of the fluorescent proteins mKO2-hCdt1 (red) and mAG1-hGem (green) of the Fucci plasmid according to the different phases of the cell cycle. (G) Coronal sections of E16.5 cerebral cortices injected with either a control, $RhoA^{DN}$, or $RhoA^{WT}$ expression plasmids at E15.5 along with Fucci plasmid. (H) Quantification of the $mAG1^+$ (S, G_2, or M phase), $mKO2^+$ (G_1 phase), or $mAG1^+$ $mKO2^+$ (S phase entry) cells. $n \geq 14$. Error bars, SEM. $***P < 0.001$, $**P < 0.01$, $*P < 0.05$; NS, not significant. Scale bar, 50 μm.

To support this observation, we electroporated E15.5 brains to express a fluorescence ubiquitination-based cell cycle reporter (FUCCI) (*71*). This fluorescent technique allows the visualization of the G_1 phase, the transition from G_1 to S, and the S-G_2-M phases (Fig. 10F). One day after the surgery, this reporter revealed a higher number of cells in the G_1 phase subsequent to RhoA GoF and a proportional decrease in the number of cells in transition to the S phase as well as in the S-G_2-M phases, compared to control brains. RhoA inhibition induced the opposite phenotype (Fig. 10, G to H).

Comparable to RhoA inhibition, the knockdown of *Mkl1* and *Mkl2*, or the expression of SRF-EnRD, induced a shortening of the G_1 phase of the cell cycle (Fig. 10D). On the other hand, the expression of MKL1 or SRF-TAD lengthened it (Fig. 10D). A mild expression of SRF-EnRD, as well as the knockdown of *Mkl1* and *Mkl2*, rescued the decrease in EdU^+ cells resulting from the RhoA-mediated G_1 phase elongation (Fig. 10E). Quite unexpectedly, MKL2 decreased the length of the G_1 phase, although it has the same effect on differentiation as RhoA, MKL1, and SRF (Fig. 10D), suggesting that differentiation and length of the G_1 phase are not

linked, at least when regulated by MKL/SRF in E15.5 embryonic brains. Supporting this conclusion, modifications of Bcl6 expression could not restore the length of the cell cycle G_1 phase affected by the modulation of RhoA activity (Fig. 10E), although we showed that it was able to rescue neurogenesis and cell positioning. Overall, these results show that RhoA, MKL1, and SRF induce a lengthening of the cell cycle G_1 phase, but this is controlled independently from neurogenesis, and this G_1 phase lengthening has no influence on neurogenesis during the investigated developmental period.

DISCUSSION

Cell positioning, progenitor proliferation, and control of neuronal production are important for the correct functioning of the neocortex. Neural cell migration and the spatiotemporal control of fate transition are regulated by the interplay of intrinsic and extrinsic signals. A key question is how these multiple pathways interact to convey a comprehensible signal for the cell.

Cross-talk between the RhoA-dependent MKL/SRF pathway and Bcl6

Here, we demonstrated that RhoA regulates apical and basal neural progenitors position and neurogenesis through the transcriptional activity of the MKL/SRF complex and inhibition of Bcl6 repressor function. Down-regulation of RhoA, MKL, or SRF during mid-neurogenic stage leads to an increased neurogenesis and to the mispositioning of RGCs, BPs, and neurons in the VZ and sVZ. Their GoF results in an accumulation of all affected cells near the apical ventricular surface and a decreased neurogenesis (a graphical model is provided in fig. S12). We also brought to light a reciprocal regulatory loop between the Bcl6 transcription repressor and MKL/SRF in vivo and in vitro. We show that Bcl6 and SRF have a direct molecular interaction and, together, form a complex with MKL1. Dimerization is crucial for MKL, SRF, or Bcl6 to associate with some of their partners and regulate gene expression (*58*, *63*, *64*, *72*). In this study, we found that Bcl6/SRF interaction inhibits Bcl6 and SRF homodimerizations but does not significantly affect the interaction between SRF and MKL or the formation of the MKL1 homodimer. Therefore, Bcl6, SRF, and MKL are likely to form an inactive complex that inhibits both pathways. These inhibitions might be partial and reduce their affinity for specific promoters. It is, however, nonexclusively possible that they form a previously unidentified complex, maybe cooperating with new partners, that induces the transcription of a different set of genes.

Bcl6 is a well-studied oncogene protein known for its significance in B cell lymphoma. To function properly, Bcl6 relies on his interactions with multiple partners such as BCL6 corepressor, nuclear receptor corepressor 1 and 2, C-terminal binding protein, and nucleosome remodeling deacetylase (*73*). Here, we found that the interaction of SRF with Bcl6 reduces Bcl6 transcriptional repressive activity both in vitro and in vivo during the development of the neocortex. This interaction with SRF may, in addition to Bcl6 homodimerization, disrupt or alter some of Bcl6 important partner interactions, influencing Bcl6 regulatory functions.

In addition, our results reveal that MKL1 is involved in both neurogenesis and cell positioning, while MKL2 affects neurogenesis only. To gain further insight, it would be of interest to identify the transcriptional profile of genes regulated by these transcriptional complexes both associated with, or independent from Bcl6. This analysis could help clarify their specific role in neurogenesis or in cell positioning and under the influence of RhoA. Many of those genes would most likely be related to signaling cascades, previously shown to be influenced by RhoA/MKL/SRF and Bcl6 in other systems, and that regulate self-renewal, neurogenesis, or cell migration (*46*, *74*–*76*). Another set of interesting genes that could be regulated are those coding for extracellular matrix (ECM) proteins. The ECM has an influence on cell behavior, proliferation, or migration of neural cells (*77*). Notably, during the development of the cerebral cortex, the extracellular environment experiences variations in its stiffness. Different regions or zones within the cortex exhibit different levels of stiffness as the brain matures and develops (*78*). It suggests that this dynamic change in the ECM stiffness may play an important role in influencing cell migration, proliferation, and differentiation, thereby contributing to the processes of neurogenesis and cell positioning. Moreover, the ECM and its stiffness can affect RhoA activity or modulate how the cells respond to RhoA activity (*79*, *80*) and modulate MKL1/SRF functions (*81*). Reciprocally, one way in which RhoA/MKL/SRF/Bcl6 interaction could affect cell migration and differentiation is through changes in the expression of ECM proteins or on the receptors sensing the ECM such as the integrins. It is therefore plausible that the ECM and the RhoA/MKL/SRF/Bcl6 pathway mutually influence each other and their effects on neurogenesis and cell positioning during neocortical development.

The interest of these findings goes beyond brain development. For instance, both Bcl6 and the MKL/SRF complex are linked to the development of cancer, and the interaction of these two pathways could lead to drug resistance (*74*, *82*). Understanding the consequences of Bcl6 cross-talk with RhoA/MKL/SRF in the brain and other organs during development and adulthood could be key to developing effective treatments for complex diseases such as developmental disorders and cancer.

Regulation of RGC neurogenic output

Our data uncovered a function of the MKL/SRF pathway in the control of neurogenesis and cell positioning. This is consistent with the development of human primary microcephaly resulting from rare genetic variation in *MKL2* (*83*). Association of the MKL/SRF pathway was also reported with autism spectrum disorders, which involve early brain malformations including defects in neurogenesis and migration (*25*, *84*).

It is tempting to link our observed effect on neurogenesis to RGCs′ IKNM because it is believed to be important for the regulation of cell fate decision (*9*). Previous studies have proposed that myosin II, a well-known RhoA downstream effector, could regulate IKNM (*85*), although this finding has been disputed (*86*). Our data suggest that RhoA might control IKNM through the expression of a set of genes regulated by the MKL1/SRF complex. However, we found that a mild increase in MKL1 or SRF, or the modulation of MKL2, triggers changes in neurogenesis with no effect on cell positioning. This suggests that, under our experimental conditions, neurogenesis and migration (including the IKNM) are separately regulated. A second argument in favor of this model is that, while the modulation of Bcl6 alone affects neurogenesis in a manner opposite to the RhoA/MKL/SRF pathway, it does not disturb cell positioning. The MKL/SRF and Bcl6 interaction is likely to regulate neurogenesis and IKNM through the control of separate sets of genes.

We also found that the length of the G_1 phase of the cell cycle is affected by RhoA and its downstream effectors MKL/SRF. Previous investigations reported a correlation between a longer G_1 phase and increased neurogenesis (*52*, *87*, *88*). The hypothesis is that a certain length of the G_1 phase is necessary to reach a sufficient accumulation of fate-determining factors driving neurogenesis. However, in our work, we found that the RhoA/MKL/SRF pathway increases G_1 length but decreases neurogenesis. In addition, Bcl6 rescues neurogenesis affected by modifications of RhoA activity without rescuing the G_1 phase defect, suggesting that cell fate decision and neurogenesis do not depend on the length of the G_1 phase in our experimental conditions. The reason for this discrepancy might be the difference in the stage investigated. The length of the G_1 phase is much longer during later stages of cortical development (*54*), and while the other groups performed their experiments during the early neurogenic phase, we focused our work during mid- to late neurogenesis. At the stage of our main investigations at E15.5, G_1 might be too long, and the reductions in G_1 phase length observed in this work are not sufficient to reach a hypothetic threshold that would be necessary to influence cell fate. Moreover, the mechanistic coupling between cell cycle length and cell fate determination could be context dependent and influenced by the specific level of RhoA/MKL/SRF activity and the preexisting molecular landscape of the studied cells, which differs in early and late RGCs. Our in vivo experiments at E13 showed a weaker effect on neurogenesis compared to E15.5, while the effect on cell positioning remained very similar. However, considering that multiple mechanisms likely collaborate to regulate neurogenesis, it is possible that the RhoA/MKL/SRF pathway overrides the effects of a longer G_1 phase through its regulation of important signals such as Shh, Wnt, Notch, and Hippo. This regulatory effect might occur through a direct action of RhoA on these pathways (*34*, *89*, *90*), or indirectly through its regulation of Bcl6, which has been shown to modify the expression of multiple targets of Shh, Wnt, and Notch (*46*).

Last, we note that the molecules studied here not only affect neurogenesis but also influence the type of neurogenesis. We observed that the activation of the RhoA/MKL/SRF pathway or the inhibition of Bcl6 has a stronger inhibitory effect on direct neurogenesis compared to its milder effect on indirect neurogenesis. It would be interesting to investigate this question at the beginning of the neurogenic phase when direct neurogenesis is more prevalent.

Regulation of BP migration

BPs are not attached to the apical surface and leave the VZ where they are generated to reach the sVZ (*91*). Little is known about the molecular mechanisms that regulate the migration of BPs. Inhibition of the adenosine triphosphate receptor P2Y1 retards BP migration, suggesting a potential involvement of Ca^{2+} signaling (*92*). It was more recently found that HDAC1 and HDAC2 temporarily regulate BPs positioning when tested at E10.5 but not at a later stage after E13.5 (*18*). Here, we provide evidence that, when tested at E13 or at E15.5, RhoA negatively regulates the migration of committed BPs away from the VZ and into the sVZ through the activation of the MKL/SRF transcription factor complex, which undergo a mutual repression with Bcl6. It is plausible that an early mechanism involving HDAC1 and 2 regulates BP migration during the very first phase of neurogenesis, while another mechanism involving RhoA/MKL/SRF and Bcl6 takes over the function.

Signaling proteins can be regulated through the modulation of their activity or of their abundance. The expression of genes associated with the actin cytoskeleton and receptors involved in cell migration is regulated by the MKL/SRF complex from *Drosophila* to mammals (*93*, *94*). Inhibition of BCL6 impairs the migration of trophoblastic cells by reducing the expression of cell adhesion molecules and compromising the dynamics of the actin cytoskeleton (*76*), which could be due to the cross-regulation of Bcl6 with MKL/SRF found in this work.

Last, while both MKL1 and MKL2 are involved in neurogenesis, only MKL1 is implicated in cell positioning. A similar difference was reported with MKL1, but not MKL2, involved in vascular smooth muscle cell migration (*95*). Determining which genes are specifically regulated by MKL1 in the developing neocortex would help further our understanding of the mechanisms involved in BP migration.

MATERIALS AND METHODS

Plasmids

pCAG:GFP (Addgene) and pCAG:Nuclear Localization Signal (NLS)-GFP (F. Kubo, National Institute of Genetics) were used for co-electroporation. RhoA, MKL1, MKL2, and SRF sequences were amplified from cDNA and inserted into the pCAG:GFP vector or the pEBG vector for GFP or glutathione *S*-transferase (GST) fusion. pEBG was a gift from D. Baltimore (Addgene, plasmid no. 22227). $RhoA^{DN}$ contains the T19N mutation. pCAGIG:Bcl6 was a gift from P. Vanderhaeghen. The Bcl6 form unable to interact with SRF contains the mutations C121F in the first SRF-binding site and P421L in the second SRF-binding site. Myc or HA tag sequences were inserted in one of the primers used for the polymerase chain reaction (PCR) amplifications before subcloning into pCAG vector. For the TriFC, the sfGFP1-9 complementary fragment, the β strand 10 (S10) and S11 of sfGFP were subcloned into the pCAG vector by PCR from UBQ10:sXVE:(MCS)-S11-DI-GFP1–9 (Addgene, plasmid no. 108260) and UBQ10-sXVE-S10-(MCS)-3xHA (Addgene, plasmid no. 108178). MKL1, MKL2, SRF, and Bcl6 were subcloned into pCAG:S10 and pCAG:S11 by PCR. Dimerization-deficient MKL1 mutant (MKL1ΔLZ) was deleted of the LZ domain by PCR using junction primers that inserted Xho I sites six residues between the codons 552 and 559 and subcloned into pCAG:GFP(S10) and pCAG:GFP(S11). pLKO.1-MKL1/2 shRNA was a gift from R. Prywes (Addgene, plasmid no. 27161). ShMKL1 and shMKL2 were constructed by insertion of the sequence GGGTAGCAGACAGTTCC TCCTTCAAGAGAGGAGGAACTGTCTGCTACCTTTTTGGA A (shMKL1) or GGCCATCCCAAGAATCCAAATTCAAGA GATTTGGATTCTTGGGATGGCTTTTTGGAA (shMKL2) using a primer annealing procedure into pSCV2 vector. pSCV2:shBcl6 was a gift from P. Vanderhaeghen (*45*). pSCV2:shBCl6(#2) was designed according to the sequence from (*45*). tFucci(SA)5 was a gift from A. Miyawaki (Addgene, plasmid no. 153520). pGL4:SRF-RE-Luc was purchased from Promega. BCl6(BS)-Luc was constructed by insertion of a single copy of the Bcl6 binding site (GAAAA TTCCTAGAAAGCATA) in front of a cytomegalovirus (CMV) promoter driving luciferase expression.

Cell culture and transfection

HEK293T, HeLa, and Neuro-2a cells were maintained in Dulbecco's modified Eagle's medium (DMEM) supplemented with 10% fetal bovine serum, 1% glutamine, and 1% penicillin-streptomycin. All cells were transfected using the PolyJet (Tebu-bio) complex according to the manufacturer's protocol.

Cell immunostaining

HeLa or HEK293T cells were spread with a density of 650,000 cells per well in a six-well plate containing three round coverslips. Twenty-four hours after transfection, the cells were gently washed three times in phosphate-buffered saline (PBS) and fixed with 3.7% paraformaldehyde (PFA, w/v) in PBS for 10 min at room temperature (RT), washed three times with PBS, permeabilized for 20 min in PBS 0.2% Triton X-100 (w/v) at RT, gently washed three times with PBS, and blocked with 1 ml of 4% normal goat serum (NGS) in PBS for 1 hour at RT. The cells were then incubated with primary antibodies diluted in blocking solution for 1 hour at RT and then washed three times with PBS. The Alexa Fluor secondary antibodies in blocking solution were added for 1 hour at RT. Last, cells were stained with 4′,6-diamidino-2-phenylindole (DAPI), washed in PBS, mounted on a slide with 20 μl of fluorescence mounting medium (DAKO), and stored at 4°C.

Co-immunoprecipitation and Western blot

HEK293T cells were seeded at 650,000 cells per 35-mm well and transfected 16 hours later with plasmid DNA using the PolyJet reagent. Twenty-four hours after transfection, the cells were gently washed three times and lysed with 300 μl of Triton X-100 lysis buffer [20 mM Hepes (pH 7.5), 150 mM NaCl, 0.5% Triton X-100, 10% glycerol, and 1 mM EDTA] protease and phosphatase inhibitor cocktail (Roche) and vigorously shaken on ice for 2 hours at 4°C. Lysates were clarified by centrifugation 10 min at 17,000*g* at 4°C.

For the coimmunoprecipitation, protein lysates were incubated with the antibody overnight at 4°C on a rotating wheel. Dynabeads protein A or G magnetic beads (Invitrogen) or glutathione resin (GenScript) agarose beads were washed three times in PBS, blocked in 500 μl of 1% bovine serum albumin/PBS for 2 hours at 4°C, washed twice with PBS, once in Triton X-100 lysis buffer, and added into the protein lysate mixture for 2 hours at 4°C on a rotating wheel. The beads were washed three times with Triton X-100 lysis buffer and boiled for 5 min in SDS loading buffer to elute the proteins. Proteins are then separated by SDS–polyacrylamide gel electrophoresis and transferred to a nitrocellulose membrane (Amersham, Biosciences) by Western blot. Membranes were blocked in 5% milk and 0.1% Tween 20 in PBS for 1 hour at RT, incubated with first antibodies overnight at 4°C, washed three times in 0.1% Tween 20 in PBS for 10 min, and incubated with horseradish peroxidase (HRP)–labeled antibodies for 1 hour at RT. Last, membranes were washed two times in 0.1% Tween 20 in PBS for 10 min at RT and once in sterile demineralized water. Chemiluminescence revelation was performed using the SuperSignal West Pico chemiluminescent substrate (Pierce) and exposed to Hyperfilm ECL (Amersham Biosciences).

Reporter gene assays

For the luciferase assay, we used the Dual-Glo Luciferase Assay System (Promega), in vitro on HEK293T lysate cells or in vivo on E16.5 cortex lysate. Luciferase activity was calculated as the Firefly activity normalized by the *Renilla* activity. Cells were spread on the well with a density of 200,000 cells per well in a 24-well plate and were transfected using the PolyJet reagent. Brains were electroporated at E15.5. All conditions were made in triplicate. Cells or cortices were collected 24 h after transfection or electroporation and lysed and dissociated in 100 μl of 1× Passive Lysis Buffer at RT for 10 min. Lysates were collected and clarified by centrifugation 5 min at 12,000 rpm and 4°C. Gene expression was measured using the Luminometer Glomax 20/20 (Promega) and the protocol "Dual-Glo" according to the manufacturer instructions.

In utero electroporation

In utero microinjection and electroporation were performed at E13 or E15.5, as previously described (*47*, *48*), using timed-pregnant CD-1 mice. Timed-pregnant mice were anesthetized using isoflurane gas, and each uterus was exposed under sterile conditions. Plasmid solutions containing 1 μg/μl of each DNA were injected into the lateral ventricles of the embryos using a heat-pulled capillary. Needles for injection were pulled from Wiretrol II glass capillaries (Drummond Scientific) and calibrated for 1-μl injections. DNA solutions were mixed in 10 mM tris (pH 8.0) with 0.01% Fast Green. Forceps-type electrodes (Nepagene) with 5-mm pads were used for electroporation (five 50-ms pulses of 45 V using the ECM830 electroporation system, Harvard Apparatus). Embryos were placed back into the abdominal cavity, and mice were sutured.

Frozen section procedure and tissue immunostaining

Whole brains were collected 23 hours after electroporation at E15.5 or at E13. GFP-positive brains were fixed for 3 hours in 3.7% PFA (w/v) in PBS solution, washed in PBS for 1 hour, and cryoprotected in a 30% sucrose in PBS solution overnight at 4°C. The brains were frozen in optimal cutting temperature compound before 6- to 10-μm-thick brain cross sections were obtained with a cryostat and placed on slides. For selected antibodies, sections were antigen-retrieved by immersion of the slides in 0.01 M sodium citrate buffer (pH 6.0) at 98°C for 20 s, cooled for 7 min at RT, and rinsed in PBS. The sections were permeabilized by being washed for 5 min in 0.2% Triton X-100 (w/v) in PBS and blocked in 4% NGS (v/v) and 0.2% Triton X-100 in PBS for 30 min at RT. Primary antibodies were incubated on slides overnight at 4°C and washed three times for 5 min in 0.2% Triton X-100 (w/v) in PBS. Alexa Fluor secondary antibodies were added for 1 hour at RT. Last, the slides were stained with DAPI and washed two times as before. The slides were coverslipped with Dako mounting medium and stored at 4°C.

EdU incorporation and tissue immunostaining

Following IUE, the operated mouse received an intraperitoneal injection with 5 μM EdU at either 8, 10, 12, 18, or 23 hours. The embryos were dissected 30 min after injection. The collected brains were fixed and sectioned as previously described. Sections were washed three times for 5 min in PBS, permeabilized in 0.2% Triton X-100 (w/v) in PBS for 30 min, and washed three times. Sections were then incubated 30 min in a working solution containing 100 mM tris-HCl, 4 mM $CuSO_4$, 2 μM sulfo-cyanine 3 azide (Lumiprobe, no. D1330), and 100 mM sodium ascorbate. The slides were then rinsed three more times in PBS for 5 min at RT before continuing with the immunostaining protocol as described previously.

Microscopy

Images for cell and cryosection staining were acquired with an Olympus FV1000 confocal microscope or a Zeiss Axio Observer A1 epifluorescence microscope.

Organotypic slice culture of cerebral cortices and time-lapse confocal microscopy

Three hundred–millimeter embryonic brain slices were prepared using a vibratome (World Precision Instruments) as previously described (*48*). In brief, the whole fetal brain was embedded in 4% low melting agarose (Promega, Madison, WI) prepared in DMEM-Hanks' F12 medium with glutamine, glucose, and Hepes and glued on a vibratome support using cyanoacrylate. The slices were cut in the coronal plane. Time-lapse confocal microscopy was performed using an Achroplan 3 20/0.50 with a Zeiss LSM 5 Pascal confocal on an Axioskop2 upright microscope. The slices were embedded in a drop of 3% agarose and cultured in a chamber on a heated stage (Warner Instruments) in DMEM-F12 (Invitrogen) supplemented with B27 (Invitrogen) and 10% serum. The medium was preheated at 37°C and equilibrated with 95% O_2 and 5% CO_2. The medium was flowed into the chamber at about 5 ml/hour. Repetitive acquisitions were performed in laterodorsal regions of the cortex in which 25 successive z optical planes spanning 120 mm were acquired. Z-stacks were selected and combined in Zeiss LSM Image Browser. Slight drifts of the slices were corrected using the ImageJ registration tool Turboreg (P. Thévenaz, Biomedical Imaging Group, Swiss Federal Institute of Technology, Lausanne, Switzerland).

Antibodies

The following antibodies were used for immunofluorescence, immunoprecipitation, or biochemistry: mouse anti–β-actin (Santa Cruz Biotechnology), mouse anti-GST (Sigma-Aldrich), mouse anti-HA.11 clone 16B12 (Covance), rabbit anti-HA.11 clone 16B12 (Eurogentec), mouse anti-Myc (Eurogentec), rabbit anti-Myc (Cell Signaling Technology), rabbit anti–α-catenin (Sigma-Aldrich), mouse anti-Ki67 (BD Biosciences), rabbit anti–phospho-Histone H3 (Cell Signaling Technology), rabbit anti-Sox2 (Millipore), mouse anti-Sox2 (Cell Signaling Technology), rabbit anti-Trb2 (Abcam), mouse anti-SatB2 (Abcam), mouse anti-Nestin (Millipore), and rabbit anti-ZO.1 (Invitrogen). Goat secondary antibodies labeled with Alexa 488, 568, and 647 (Invitrogen) were used for immunofluorescence. Goat anti-mouse or anti-rabbit HRP-conjugated secondary antibodies (Cell Signaling Technology) were used for biochemistry.

Measurement of cell positioning and differentiation in histological sections

For E16.5 brain, the height of the whole cerebral wall was divided into 40 bins, but only the first 20 bins were included in the analysis since no cells were observed above them in any of our experiments. For E14 brains, the height of the whole cerebral wall was divided into 20 bins, and all of the bins were included in the analysis. Counting was performed by densitometry using ImageJ. To give similar importance to low-fluorescence and high-fluorescence cells, the pictures were first modified into binary images to equalize the brightness of each positive cell. Then, the area covered by the pixels was quantified in ImageJ for each bin.

Measurement of protein interaction in TriFC

Five pictures per coverslip were taken for each condition to cover the whole area of transfected cells. Using QuPath, we measured the total number of $DAPI^+$ cells. Each picture includes about 300 cells. Using ImageJ, we quantified the pixels positive for the GFP fluorescence in each picture. The GFP fluorescence was then divided by the number of cells measured per picture.

Statistical analysis

Statistical significance was determined using two-tailed, unpaired Student's *t* tests for two-population comparisons or one-way analysis of variance (ANOVA) followed by Bonferroni's post hoc test for multiple comparisons across *N* samples, where *N* is the number of embryos or experiments as defined in the figure legends. Percentage values were modified with arcsin transformations before applying statistical tests.

Mice

CD1 mice were bred in standard conditions and animal procedures were carried out in accordance with European guidelines and approved by the animal ethics committee of the Université catholique de Louvain. Approval number is 2021/UCL/MD/029.

Key resources table

Key resources are provided in table S1.

Supplementary Materials

This PDF file includes:
Figs. S1 to S12
Legends for movies S1 and S2
Table S1

Other Supplementary Material for this manuscript includes the following:
Movies S1 and S2

REFERENCES AND NOTES

1. N. Gaspard, P. Vanderhaeghen, Laminar fate specification in the cerebral cortex. *F1000 Biol. Rep.* **3**, 6 (2011).
2. Y. Lin, J. Yang, Z. Shen, J. Ma, B. D. Simons, S. H. Shi, Behavior and lineage progression of neural progenitors in the mammalian cortex. *Curr. Opin. Neurobiol.* **66**, 144–157 (2021).
3. P. Gao, M. P. Postiglione, T. G. Krieger, L. Hernandez, C. Wang, Z. Han, C. Streicher, E. Papusheva, R. Insolera, K. Chugh, O. Kodish, K. Huang, B. D. Simons, L. Luo, S. Hippenmeyer, S. H. Shi, Deterministic progenitor behavior and unitary production of neurons in the neocortex. *Cell* **159**, 775–788 (2014).
4. E. Kon, A. Cossard, Y. Jossin, Neuronal polarity in the embryonic mammalian cerebral cortex. *Front. Cell. Neurosci.* **11**, 163 (2017).
5. A. Kriegstein, A. Alvarez-Buylla, The glial nature of embryonic and adult neural stem cells. *Annu. Rev. Neurosci.* **32**, 149–184 (2009).
6. L. Pinto, M. T. Mader, M. Irmler, M. Gentilini, F. Santoni, D. Drechsel, R. Blum, R. Stahl, A. Bulfone, P. Malatesta, J. Beckers, M. Gotz, Prospective isolation of functionally distinct radial glial subtypes--lineage and transcriptome analysis. *Mol. Cell. Neurosci.* **38**, 15–42 (2008).
7. W. B. Huttner, Y. Kosodo, Symmetric versus asymmetric cell division during neurogenesis in the developing vertebrate central nervous system. *Curr. Opin. Cell Biol.* **17**, 648–657 (2005).
8. E. Taverna, W. B. Huttner, Neural progenitor nuclei IN motion. *Neuron* **67**, 906–914 (2010).
9. P. C. Spear, C. A. Erickson, Interkinetic nuclear migration: A mysterious process in search of a function. *Dev. Growth Differ.* **54**, 306–316 (2012).
10. Y. Jossin, Molecular mechanisms of cell polarity in a range of model systems and in migrating neurons. *Mol. Cell. Neurosci.* **106**, 103503 (2020).

11. T. Sun, R. F. Hevner, Growth and folding of the mammalian cerebral cortex: From molecules to malformations. *Nat. Rev. Neurosci.* **15**, 217–232 (2014).
12. X. Lv, S. Q. Ren, X. J. Zhang, Z. Shen, T. Ghosh, A. Xianyu, P. Gao, Z. Li, S. Lin, Y. Yu, Q. Zhang, M. Groszer, S. H. Shi, TBR2 coordinates neurogenesis expansion and precise microcircuit organization via protocadherin 19 in the mammalian cortex. *Nat. Commun.* **10**, 3946 (2019).
13. A. B. Mihalas, R. F. Hevner, Clonal analysis reveals laminar fate multipotency and daughter cell apoptosis of mouse cortical intermediate progenitors. *Development* **145**, dev164335 (2018).
14. N. A. Vasistha, F. García-Moreno, S. Arora, A. F. Cheung, S. J. Arnold, E. J. Robertson, Z. Molnár, Cortical and clonal contribution of Tbr2 expressing progenitors in the developing mouse brain. *Cereb. Cortex* **25**, 3290–3302 (2015).
15. V. Martínez-Cerdeño, S. C. Noctor, A. R. Kriegstein, The role of intermediate progenitor cells in the evolutionary expansion of the cerebral cortex. *Cereb. Cortex* **16**Suppl 1, i152–i161 (2006).
16. S. C. Noctor, V. Martinez-Cerdeno, A. R. Kriegstein, Distinct behaviors of neural stem and progenitor cells underlie cortical neurogenesis. *J. Comp. Neurol.* **508**, 28–44 (2008).
17. B. Libe-Philippot, P. Vanderhaeghen, Cellular and molecular mechanisms linking human cortical development and evolution. *Annu. Rev. Genet.* **55**, 555–581 (2021).
18. T. Tang, Y. Zhang, Y. Wang, Z. Cai, Z. Lu, L. Li, R. Huang, A. Hagelkruys, P. Matthias, H. Zhang, C. Seiser, Y. Xie, HDAC1 and HDAC2 regulate intermediate progenitor positioning to safeguard neocortical development. *Neuron* **101**, 1117–1133.e5 (2019).
19. C. G. Woods, Human microcephaly. *Curr. Opin. Neurobiol.* **14**, 112–117 (2004).
20. A. Contestabile, F. Benfenati, L. Gasparini, Communication breaks-down: From neurodevelopment defects to cognitive disabilities in Down syndrome. *Prog. Neurobiol.* **91**, 1–22 (2010).
21. F. Francis, G. Meyer, C. Fallet-Bianco, S. Moreno, C. Kappeler, A. C. Socorro, F. P. Tuy, C. Beldjord, J. Chelly, Human disorders of cortical development: From past to present. *Eur. J. Neurosci.* **23**, 877–893 (2006).
22. T. J. Raedler, M. B. Knable, D. R. Weinberger, Schizophrenia as a developmental disorder of the cerebral cortex. *Curr. Opin. Neurobiol.* **8**, 157–161 (1998).
23. L. de Nijs, N. Wolkoff, B. Coumans, A. V. Delgado-Escueta, T. Grisar, B. Lakaye, Mutations of EFHC1, linked to juvenile myoclonic epilepsy, disrupt radial and tangential migrations during brain development. *Hum. Mol. Genet.* **21**, 5106–5117 (2012).
24. O. Penagarikano, B. S. Abrahams, E. I. Herman, K. D. Winden, A. Gdalyahu, H. Dong, L. I. Sonnenblick, R. Gruver, J. Almajano, A. Bragin, P. Golshani, J. T. Trachtenberg, E. Peles, D. H. Geschwind, Absence of CNTNAP2 leads to epilepsy, neuronal migration abnormalities, and core autism-related deficits. *Cell* **147**, 235–246 (2011).
25. R. Stoner, M. L. Chow, M. P. Boyle, S. M. Sunkin, P. R. Mouton, S. Roy, A. Wynshaw-Boris, S. A. Colamarino, E. S. Lein, E. Courchesne, Patches of disorganization in the neocortex of children with autism. *N. Engl. J. Med.* **370**, 1209–1219 (2014).
26. M. C. Manzini, C. A. Walsh, What disorders of cortical development tell us about the cortex: One plus one does not always make two. *Curr. Opin. Genet. Dev.* **21**, 333–339 (2011).
27. A. L. Gompers, L. Su-Feher, J. Ellegood, N. A. Copping, M. A. Riyadh, T. W. Stradleigh, M. C. Pride, M. D. Schaffler, A. A. Wade, R. Catta-Preta, I. Zdilar, S. Louis, G. Kaushik, B. J. Mannion, I. Plajzer-Frick, V. Afzal, A. Visel, L. A. Pennacchio, D. E. Dickel, J. P. Lerch, J. N. Crawley, K. S. Zarbalis, J. L. Silverman, A. S. Nord, Germline Chd8 haploinsufficiency alters brain development in mouse. *Nat. Neurosci.* **20**, 1062–1073 (2017).
28. A. M. M. Sousa, K. A. Meyer, G. Santpere, F. O. Gulden, N. Sestan, Evolution of the human nervous system function, structure, and development. *Cell* **170**, 226–247 (2017).
29. C. Olenik, K. Aktories, D. K. Meyer, Differential expression of the small GTP-binding proteins RhoA, RhoB, Cdc_{42}u and Cdc_{42}b in developing rat neocortex. *Brain Res. Mol. Brain Res.* **70**, 9–17 (1999).
30. A. X. Liu, N. Rane, J. P. Liu, G. C. Prendergast, RhoB is dispensable for mouse development, but it modifies susceptibility to tumor formation as well as cell adhesion and growth factor signaling in transformed cells. *Mol. Cell. Biol.* **21**, 6906–6912 (2001).
31. A. Hakem, O. Sanchez-Sweatman, A. You-Ten, G. Duncan, A. Wakeham, R. Khokha, T. W. Mak, RhoC is dispensable for embryogenesis and tumor initiation but essential for metastasis. *Genes Dev.* **19**, 1974–1979 (2005).
32. S. Cappello, C. R. Bohringer, M. Bergami, K. K. Conzelmann, A. Ghanem, G. S. Tomassy, P. Arlotta, M. Mainardi, M. Allegra, M. Caleo, J. van Hengel, C. Brakebusch, M. Götz, A radial glia-specific role of RhoA in double cortex formation. *Neuron* **73**, 911–924 (2012).
33. D. Herzog, P. Loetscher, J. van Hengel, S. Knusel, C. Brakebusch, V. Taylor, U. Suter, J. B. Relvas, The small GTPase RhoA is required to maintain spinal cord neuroepithelium organization and the neural stem cell pool. *J. Neurosci.* **31**, 5120–5130 (2011).
34. K. Katayama, J. Melendez, J. M. Baumann, J. R. Leslie, B. K. Chauhan, N. Nemkul, R. A. Lang, C. Y. Kuan, Y. Zheng, Y. Yoshida, Loss of RhoA in neural progenitor cells causes the disruption of adherens junctions and hyperproliferation. *Proc. Natl. Acad. Sci. U.S.A.* **108**, 7607–7612 (2011).
35. J. Bonnefont, P. Vanderhaeghen, Neuronal fate acquisition and specification: Time for a change. *Curr. Opin. Neurobiol.* **66**, 195–204 (2021).
36. A. C. O'Neill, C. Kyrousi, J. Klaus, R. J. Leventer, E. P. Kirk, A. Fry, D. T. Pilz, T. Morgan, Z. A. Jenkins, M. Drukker, S. F. Berkovic, I. E. Scheffer, R. Guerrini, D. M. Markie, M. Götz, S. Cappello, S. P. Robertson, A primate-specific isoform of PLEKHG6 regulates neurogenesis and neuronal migration. *Cell Rep.* **25**, 2729–2741.e6 (2018).
37. S. A. Lambert, A. Jolma, L. F. Campitelli, P. K. Das, Y. Yin, M. Albu, X. Chen, J. Taipale, T. R. Hughes, M. T. Weirauch, The human transcription factors. *Cell* **172**, 650–665 (2018).
38. G. Posern, R. Treisman, Actin' together: Serum response factor, its cofactors and the link to signal transduction. *Trends Cell Biol.* **16**, 588–596 (2006).
39. S. Arsenian, B. Weinhold, M. Oelgeschlager, U. Ruther, A. Nordheim, Serum response factor is essential for mesoderm formation during mouse embryogenesis. *EMBO J.* **17**, 6289–6299 (1998).
40. E. M. Pinheiro, Z. Xie, A. L. Norovich, M. Vidaki, L. H. Tsai, F. B. Gertler, Lpd depletion reveals that SRF specifies radial versus tangential migration of pyramidal neurons. *Nat. Cell Biol.* **13**, 989–995 (2011).
41. C. Stritt, B. Knöll, Serum response factor regulates hippocampal lamination and dendrite development and is connected with reelin signaling. *Mol. Cell. Biol.* **30**, 1828–1837 (2010).
42. M. Scandaglia, E. Benito, C. Morenilla-Palao, A. Fiorenza, B. Del Blanco, Y. Coca, E. Herrera, A. Barco, Fine-tuned SRF activity controls asymmetrical neuronal outgrowth: Implications for cortical migration, neural tissue lamination and circuit assembly. *Sci. Rep.* **5**, 17470 (2015).
43. P. P. Y. Lu, N. Ramanan, Serum response factor is required for cortical axon growth but is dispensable for neurogenesis and neocortical lamination. *J. Neurosci.* **31**, 16651–16664 (2011).
44. H. Liang, S. Hippenmeyer, H. T. Ghashghaei, A Nestin-cre transgenic mouse is insufficient for recombination in early embryonic neural progenitors. *Biol. Open* **1**, 1200–1203 (2012).
45. L. Tiberi, J. van den Ameele, J. Dimidschstein, J. Piccirilli, D. Gall, A. Herpoel, A. Bilheu, J. Bonnefont, M. Iacovino, M. Kyba, T. Bouschet, P. Vanderhaeghen, BCL6 controls neurogenesis through Sirt1-dependent epigenetic repression of selective Notch targets. *Nat. Neurosci.* **15**, 1627–1635 (2012).
46. J. Bonnefont, L. Tiberi, J. van den Ameele, D. Potier, Z. B. Gaber, X. Lin, A. Bilheu, A. Herpoel, F. D. Velez Bravo, F. Guillemot, S. Aerts, P. Vanderhaeghen, Cortical neurogenesis requires Bcl6-mediated transcriptional repression of multiple self-renewal-promoting extrinsic pathways. *Neuron* **103**, 1096–1108.e4 (2019).
47. H. Tabata, K. Nakajima, Efficient in utero gene transfer system to the developing mouse brain using electroporation: Visualization of neuronal migration in the developing cortex. *Neuroscience* **103**, 865–872 (2001).
48. Y. Jossin, J. A. Cooper, Reelin, Rap1 and N-cadherin orient the migration of multipolar neurons in the developing neocortex. *Nat. Neurosci.* **14**, 697–703 (2011).
49. T. Miyata, Asymmetric cell division during brain morphogenesis. *Prog. Mol. Subcell. Biol.* **45**, 121–142 (2007).
50. T. Miyata, A. Kawaguchi, K. Saito, M. Kawano, T. Muto, M. Ogawa, Asymmetric production of surface-dividing and non-surface-dividing cortical progenitor cells. *Development* **131**, 3133–3145 (2004).
51. E. K. Stancik, I. Navarro-Quiroga, R. Sellke, T. F. Haydar, Heterogeneity in ventricular zone neural precursors contributes to neuronal fate diversity in the postnatal neocortex. *J. Neurosci.* **30**, 7028–7036 (2010).
52. L. J. Pilaz, D. Patti, G. Marcy, E. Ollier, S. Pfister, R. J. Douglas, M. Betizeau, E. Gautier, V. Cortay, N. Doerflinger, H. Kennedy, C. Dehay, Forced G1-phase reduction alters mode of division, neuron number, and laminar phenotype in the cerebral cortex. *Proc. Natl. Acad. Sci. U.S.A.* **106**, 21924–21929 (2009).
53. Y. Arai, J. N. Pulvers, C. Haffner, B. Schilling, I. Nusslein, F. Calegari, W. B. Huttner, Neural stem and progenitor cells shorten S-phase on commitment to neuron production. *Nat. Commun.* **2**, 154 (2011).
54. T. Takahashi, R. S. Nowakowski, V. S. Caviness Jr., The cell cycle of the pseudostratified ventricular epithelium of the embryonic murine cerebral wall. *J. Neurosci.* **15**, 6046–6057 (1995).
55. V. Perovic, N. Sumonja, B. Gemovic, E. Toska, S. G. Roberts, N. Veljkovic, TRI_tool: A web-tool for prediction of protein-protein interactions in human transcriptional regulation. *Bioinformatics* **33**, 289–291 (2017).
56. L. Tiberi, J. Bonnefont, J. van den Ameele, S. D. Le Bon, A. Herpoel, A. Bilheu, B. W. Baron, P. Vanderhaeghen, A BCL6/BCOR/SIRT1 complex triggers neurogenesis and suppresses medulloblastoma by repressing Sonic hedgehog signaling. *Cancer Cell* **26**, 797–812 (2014).
57. S. Cabantous, H. B. Nguyen, J. D. Pedelacq, F. Koraichi, A. Chaudhary, K. Ganguly, M. A. Lockard, G. Favre, T. C. Terwilliger, G. S. Waldo, A new protein-protein interaction sensor based on tripartite split-GFP association. *Sci. Rep.* **3**, 2854 (2013).

58. F. Reed, S. T. Larsuel, M. Y. Mayday, V. Scanlon, D. S. Krause, MRTFA: A critical protein in normal and malignant hematopoiesis and beyond. *J. Biol. Chem.* **296**, 100543 (2021).
59. N. Kawamata, T. Miki, K. Ohashi, K. Suzuki, T. Fukuda, S. Hirosawa, N. Aoki, Recognition DNA sequence of a novel putative transcription factor, BCL6. *Biochem. Biophys. Res. Commun.* **204**, 366–374 (1994).
60. C. C. Chang, B. H. Ye, R. S. Chaganti, R. Dalla-Favera, BCL-6, a POZ/zinc-finger protein, is a sequence-specific transcriptional repressor. *Proc. Natl. Acad. Sci. U.S.A.* **93**, 6947–6952 (1996).
61. P. Dhordain, O. Albagli, R. J. Lin, S. Ansieau, S. Quief, A. Leutz, J. P. Kerckaert, R. M. Evans, D. Leprince, Corepressor SMRT binds the BTB/POZ repressing domain of the LAZ3/BCL6 oncoprotein. *Proc. Natl. Acad. Sci. U.S.A.* **94**, 10762–10767 (1997).
62. M. K. Vartiainen, S. Guettler, B. Larijani, R. Treisman, Nuclear actin regulates dynamic subcellular localization and activity of the SRF cofactor MAL. *Science* **316**, 1749–1752 (2007).
63. L. Pellegrini, S. Tan, T. J. Richmond, Structure of serum response factor core bound to DNA. *Nature* **376**, 490–498 (1995).
64. K. F. Ahmad, A. Melnick, S. Lax, D. Bouchard, J. Liu, C. L. Kiang, S. Mayer, S. Takahashi, J. D. Licht, G. G. Prive, Mechanism of SMRT corepressor recruitment by the BCL6 BTB domain. *Mol. Cell* **12**, 1551–1564 (2003).
65. W. H. Ernst, R. Janknecht, M. A. Cahill, A. Nordheim, Transcriptional repression mediated by the serum response factor. *FEBS Lett.* **357**, 45–49 (1995).
66. F. E. Johansen, R. Prywes, Identification of transcriptional activation and inhibitory domains in serum response factor (SRF) by using GAL4-SRF constructs. *Mol. Cell. Biol.* **13**, 4640–4647 (1993).
67. H. Niu, B. H. Ye, R. Dalla-Favera, Antigen receptor signaling induces MAP kinase-mediated phosphorylation and degradation of the BCL-6 transcription factor. *Genes Dev.* **12**, 1953–1961 (1998).
68. L. M. Mendez, J. M. Polo, J. J. Yu, M. Krupski, B. B. Ding, A. Melnick, B. H. Ye, CtBP is an essential corepressor for BCL6 autoregulation. *Mol. Cell. Biol.* **28**, 2175–2186 (2008).
69. B. D. Lee, M. R. Kim, M. Y. Kang, J. Y. Cha, S. H. Han, G. M. Nawkar, Y. Sakuraba, S. Y. Lee, T. Imaizumi, C. R. McClung, W. Y. Kim, N. C. Paek, The F-box protein FKF1 inhibits dimerization of COP1 in the control of photoperiodic flowering. *Nat. Commun.* **8**, 2259 (2017).
70. N. Watanabe, R. Kageyama, T. Ohtsuka, Hbp1 regulates the timing of neuronal differentiation during cortical development by controlling cell cycle progression. *Development* **142**, 2278–2290 (2015).
71. A. Sakaue-Sawano, M. Yo, N. Komatsu, T. Hiratsuka, T. Kogure, T. Hoshida, N. Goshima, M. Matsuda, H. Miyoshi, A. Miyawaki, Genetically encoded tools for optical dissection of the mammalian cell cycle. *Mol. Cell* **68**, 626–640.e5 (2017).
72. A. Huet, A. Parlakian, M. C. Arnaud, J. M. Glandieres, P. Valat, S. Fermandjian, D. Paulin, B. Alpert, C. Zentz, Mechanism of binding of serum response factor to serum response element. *FEBS J.* **272**, 3105–3119 (2005).
73. H. Yang, M. R. Green, Epigenetic programing of B-cell lymphoma by BCL6 and its genetic deregulation. *Front. Cell Dev. Biol.* **7**, 272 (2019).
74. R. J. Whitson, A. Lee, N. M. Urman, A. Mirza, C. Y. Yao, A. S. Brown, J. R. Li, G. Shankar, M. A. Fry, S. X. Atwood, E. Y. Lee, S. T. Hollmig, S. Z. Aasi, K. Y. Sarin, M. P. Scott, E. H. Epstein Jr., J. Y. Tang, A. E. Oro, Non-canonical hedgehog pathway activation through SRF-MKL1 promotes drug-resistance in basal cell carcinomas. *Nat. Med.* **24**, 271–281 (2018).
75. D. Gau, P. Roy, SRF'ing and SAP'ing—The role of MRTF proteins in cell migration. *J. Cell Sci.* **131**, jcs218222 (2018).
76. A. Ritter, B. K. Safdar, B. Jasmer, N. N. Kreis, A. Friemel, S. Roth, C. Solbach, F. Louwen, J. Yuan, The function of oncogene B-cell lymphoma 6 in the regulation of the migration and invasion of trophoblastic cells. *Int. J. Mol. Sci.* **21**, 8393 (2020).
77. S. Amin, V. Borrell, The extracellular matrix in the evolution of cortical development and folding. *Front. Cell Dev. Biol.* **8**, 604448 (2020).
78. M. Iwashita, N. Kataoka, K. Toida, Y. Kosodo, Systematic profiling of spatiotemporal tissue and cellular stiffness in the developing brain. *Development* **141**, 3793–3798 (2014).
79. S. M. Lim, B. A. Kreipe, J. Trzeciakowski, L. Dangott, A. Trache, Extracellular matrix effect on RhoA signaling modulation in vascular smooth muscle cells. *Exp. Cell Res.* **316**, 2833–2848 (2010).
80. C. Collins, L. D. Osborne, C. Guilluy, Z. Chen, E. T. O'Brien 3rd, J. S. Reader, K. Burridge, R. Superfine, E. Tzima, Haemodynamic and extracellular matrix cues regulate the mechanical phenotype and stiffness of aortic endothelial cells. *Nat. Commun.* **5**, 3984 (2014).
81. M. K. Willer, C. W. Carroll, Substrate stiffness-dependent regulation of the SRF-Mkl1 co-activator complex requires the inner nuclear membrane protein Emerin. *J. Cell Sci.* **130**, 2111–2118 (2017).
82. B. H. Ye, F. Lista, F. Lo Coco, D. M. Knowles, K. Offit, R. S. Chaganti, R. Dalla-Favera, Alterations of a zinc finger-encoding gene, BCL-6, in diffuse large-cell lymphoma. *Science* **262**, 747–750 (1993).
83. E. I. Ramos, G. A. Bien-Willner, J. Li, A. E. Hughes, J. Giacalone, S. Chasnoff, S. Kulkarni, M. Parmacek, F. S. Cole, T. E. Druley, Genetic variation in MKL2 and decreased downstream PCTAIRE1 expression in extreme, fatal primary human microcephaly. *Clin. Genet.* **85**, 423–432 (2014).
84. R. Holt, G. Barnby, E. Maestrini, E. Bacchelli, D. Brocklebank, I. Sousa, E. J. Mulder, K. Kantojarvi, I. Jarvela, S. M. Klauck, F. Poustka, A. J. Bailey, A. P. Monaco; EU Autism MOLGEN Consortium, Linkage and candidate gene studies of autism spectrum disorders in European populations. *Eur. J. Hum. Genet.* **18**, 1013–1019 (2010).
85. J. Schenk, M. Wilsch-Brauninger, F. Calegari, W. B. Huttner, Myosin II is required for interkinetic nuclear migration of neural progenitors. *Proc. Natl. Acad. Sci. U.S.A.* **106**, 16487–16492 (2009).
86. J. W. Tsai, W. N. Lian, S. Kemal, A. R. Kriegstein, R. B. Vallee, Kinesin 3 and cytoplasmic dynein mediate interkinetic nuclear migration in neural stem cells. *Nat. Neurosci.* **13**, 1463–1471 (2010).
87. F. Calegari, W. B. Huttner, An inhibition of cyclin-dependent kinases that lengthens, but does not arrest, neuroepithelial cell cycle induces premature neurogenesis. *J. Cell Sci.* **116**, 4947–4955 (2003).
88. C. Lange, W. B. Huttner, F. Calegari, Cdk4/cyclinD1 overexpression in neural stem cells shortens G1, delays neurogenesis, and promotes the generation and expansion of basal progenitors. *Cell Stem Cell* **5**, 320–331 (2009).
89. K. Schlessinger, A. Hall, N. Tolwinski, Wnt signaling pathways meet Rho GTPases. *Genes Dev.* **23**, 265–277 (2009).
90. O. M. Yu, S. Miyamoto, J. H. Brown, Myocardin-related transcription factor a and yes-associated protein exert dual control in G protein-coupled receptor- and RhoA-mediated transcriptional regulation and cell proliferation. *Mol. Cell. Biol.* **36**, 39–49 (2016).
91. T. Kowalczyk, A. Pontious, C. Englund, R. A. Daza, F. Bedogni, R. Hodge, A. Attardo, C. Bell, W. B. Huttner, R. F. Hevner, Intermediate neuronal progenitors (basal progenitors) produce pyramidal-projection neurons for all layers of cerebral cortex. *Cereb. Cortex* **19**, 2439–2450 (2009).
92. X. Liu, K. Hashimoto-Torii, M. Torii, T. F. Haydar, P. Rakic, The role of ATP signaling in the migration of intermediate neuronal progenitors to the neocortical subventricular zone. *Proc. Natl. Acad. Sci. U.S.A.* **105**, 11802–11807 (2008).
93. K. Somogyi, P. Rørth, Evidence for tension-based regulation of Drosophila MAL and SRF during invasive cell migration. *Dev. Cell* **7**, 85–93 (2004).
94. S. Alberti, S. M. Krause, O. Kretz, U. Philippar, T. Lemberger, E. Casanova, F. F. Wiebel, H. Schwarz, M. Frotscher, G. Schütz, A. Nordheim, Neuronal migration in the murine rostral migratory stream requires serum response factor. *Proc. Natl. Acad. Sci. U.S.A.* **102**, 6148–6153 (2005).
95. M. C. Smith, C. A. Hudson, T. E. Kimura, S. J. White, G. B. Sala-Newby, A. C. Newby, M. Bond, Divergent regulation of actin dynamics and megakaryoblastic leukemia-1 and -2 (Mkl1/2) by cAMP in endothelial and smooth muscle cells. *Sci. Rep.* **7**, 3681 (2017).

Acknowledgments: We are very grateful to J. Cooper and P. Vanderhaeghen for helpful comments on the manuscript and for sharing reagents. We thank L. Buck for the use of confocal microscope and C. Bouzin and G. Courtoy from the imaging facility for help in image analysis. We wish to thank members of the Jossin laboratory for encouragement. **Funding:** This work was supported by grants J.0129.15, J.0179.16, T.0243.18, and J.0101.21 from the Belgian National Funding for Scientific Research (FNRS). Y.J. is an FNRS investigator. A.C. was supported by a Fonds pour la Formation à la Recherche dans l'Industrie et dans l'Agriculture (FRIA) fellowship. K.S. is supported by a Special Funds for Research (FSR) fellowship. The Belgian University Foundation provided financial support to partially cover the publication fee of this manuscript. **Author contributions:** Y.J. conceptualized and supervised the study. Y.J. designed the experiments with inputs from the other authors. A.C., K.S., A.S., and Y.J. performed experiments. A.C. performed the histological and cytological studies, coimmunoprecipitation, tri-molecular fluorescence complementation, luciferase assays, plasmid constructions, and statistical analysis with the help from K.S., A.S., and Y.J. Y.J. performed the in utero electroporations and time-lapse videomicroscopy. Y.J. wrote and revised the manuscript with input from A.C. **Competing interests:** The authors declare that they have no competing interests. **Data and materials availability:** All data needed to evaluate the conclusions in the paper are present in the paper and/or the Supplementary Materials.

Submitted 18 May 2022
Accepted 16 October 2023
Published 15 November 2023
10.1126/sciadv.add0676
