# Supplementary material for: MKL/SRF and Bcl6 mutual transcriptional repression safeguards the fate and positioning of neocortical progenitor cells mediated by RhoA
Source: Sci Adv. 2023 Nov 15;9(46):eadd0676. doi: 10.1126/sciadv.add0676 (PMC10651131; doi:10.1126/sciadv.add0676)
Supplement: Supplementary file 2 — Figs. S1 to S12 Legends for movies S1 and S2 Table S1 [file sciadv.add0676_sm.pdf]

Science Advances
AAAS

Supplementary Materials for

MKL/SRF and Bcl6 mutual transcriptional repression safeguards the fate and positioning of neocortical progenitor cells mediated by RhoA

Alexia Cossard *et al.*

Corresponding author: Yves Jossin, yves.jossin@uclouvain.be

Sci. Adv. **9**, eadd0676 (2023)
DOI: 10.1126/sciadv.add0676

The PDF file includes:

Figs. S1 to S12
Legends for movies S1 and S2
Table S1

Other Supplementary Material for this manuscript includes the following:

Movies S1 and S2

Supplementary Figures

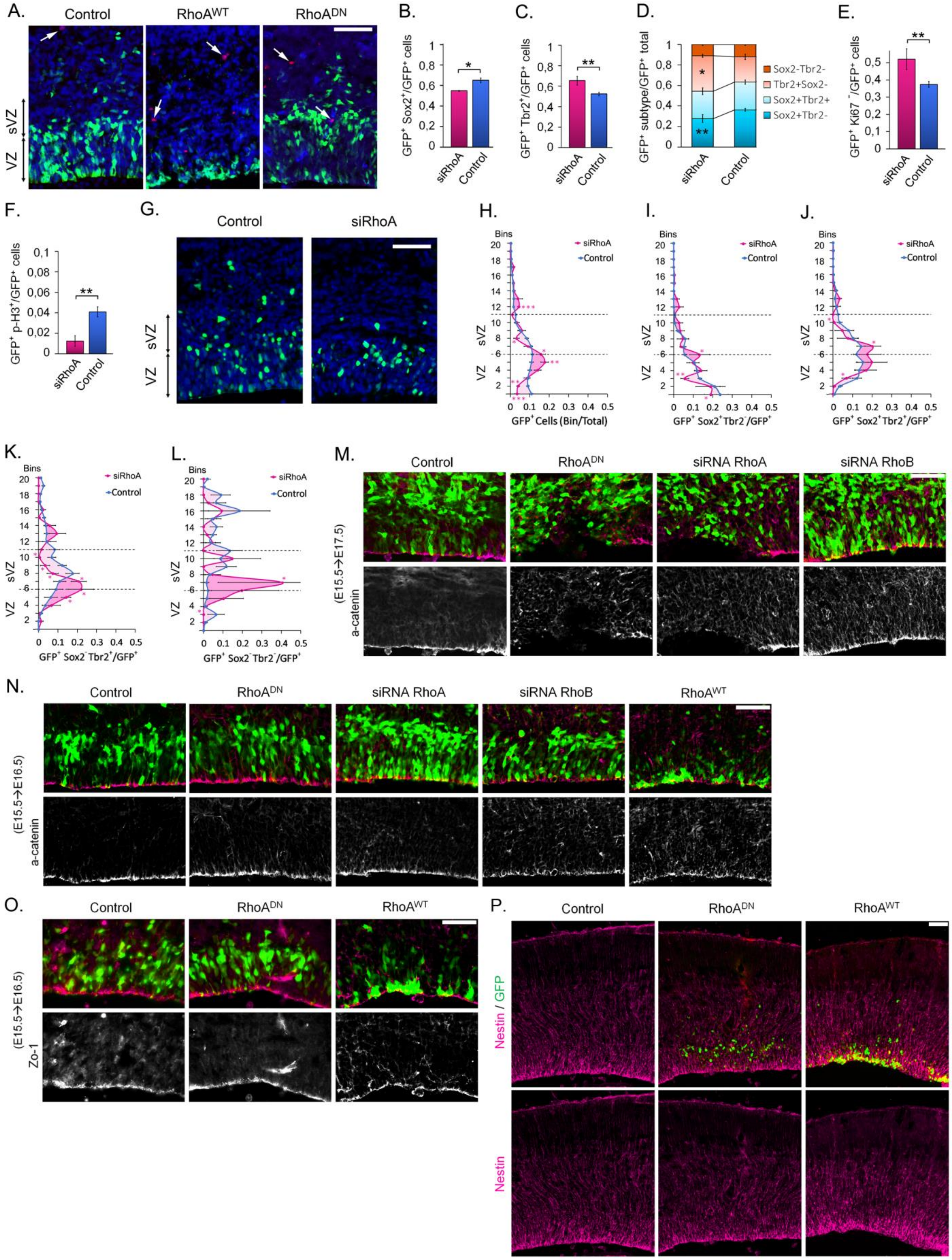

Fig. S1: RhoA regulates neurogenesis and neural cells positioning.

Embryonic brains were *in utero* electroporated at E15.5 and processed 23 hours later for immunohistological labelling. **(A)** Olig2 staining (red). Arrows shows the few Olig2+ cells. **(B-L):** siRhoA induced a similar phenotype on neurogenesis and cell positioning as $RhoA^{DN}$. **(B,C,E,F)** Quantification of double staining for (**B**) GFP^+Sox2^+, (**C**) GFP^+Tbr2^+, (**E**) GFP^+Ki67^- (cell cycle exit), and (**F**) GFP^+p-$H3^+$ (mitotic index) cells. (**B**) Control n=19 out of 13 IUE (n=19//13); siRhoA n=4//3; (**C**) Control n=21//14; siRhoA n=4//3; (**E**) Control n=12//9; siRhoA n=3//3; (**F**) Control n=7//4; siRhoA n=3//3; **(D)** Quantification of the triple staining GFP^+Sox2^+ $Tbr2^-$ (RGCs), $GFP^+Sox2^+Tbr2^+$ (Committed BPs), $GFP^+Sox2^-Tbr2^+$ (BPs), $GFP^+Sox2^-Tbr2^-$ (neurons) cells. Control n=9//5; siRhoA n=4//3. **(H-L)** The graphics indicate the proportion of cells in each bin of (**H**) GFP+ cells or (**I**) GFP^+Sox2^+ $Tbr2^-$ (RGCs), (**J**) $GFP^+Sox2^+Tbr2^+$ (Committed BPs), (**K**) $GFP^+Sox2^-Tbr2^+$ (BPs), and (**L**) $GFP^+Sox2^-Tbr2^-$ (neurons) for control, siRhoA expression vectors electroporated at E15.5 and observed at E16.5. (**H**) Control n=21//15; siRhoA n=5//3; (**I-L**) Control n=9//5; siRhoA n=5//3. **(M-O)**: The effect of RhoA downregulation on AJs stability is obvious two days after knockdown but not noticeable after 24 hours: Coronal sections of (**M**) E17.5 or (**N,O**) E16.5 murine cerebral cortices, electroporated at E15.5 for the expression of siRNA targeting either RhoA or RhoB, or for the expression of a dominant-negative form of RhoA ($RhoA^{DN}$) or of the wild-type RhoA ($RhoA^{WT}$) along with NLS-GFP (Green) and stained (Red) for (**M,N**) α-catenin or (**O**) ZO.1. **(P)**: RhoA inhibition or GoF does not disturb radial glia fibers 24 hours after surgery. Coronal sections of E16.5 murine cerebral cortices, non-electroporated (Control) or electroporated at E15.5 for the expression of GFP (Green) along with a dominant-negative form of RhoA ($RhoA^{DN}$) or the wild-type RhoA ($RhoA^{WT}$) and stained for Nestin (Red). Error bars, s.e.m., ***p<0.001, **p<0.01, *p<0.05, Scale bars: 50µm.

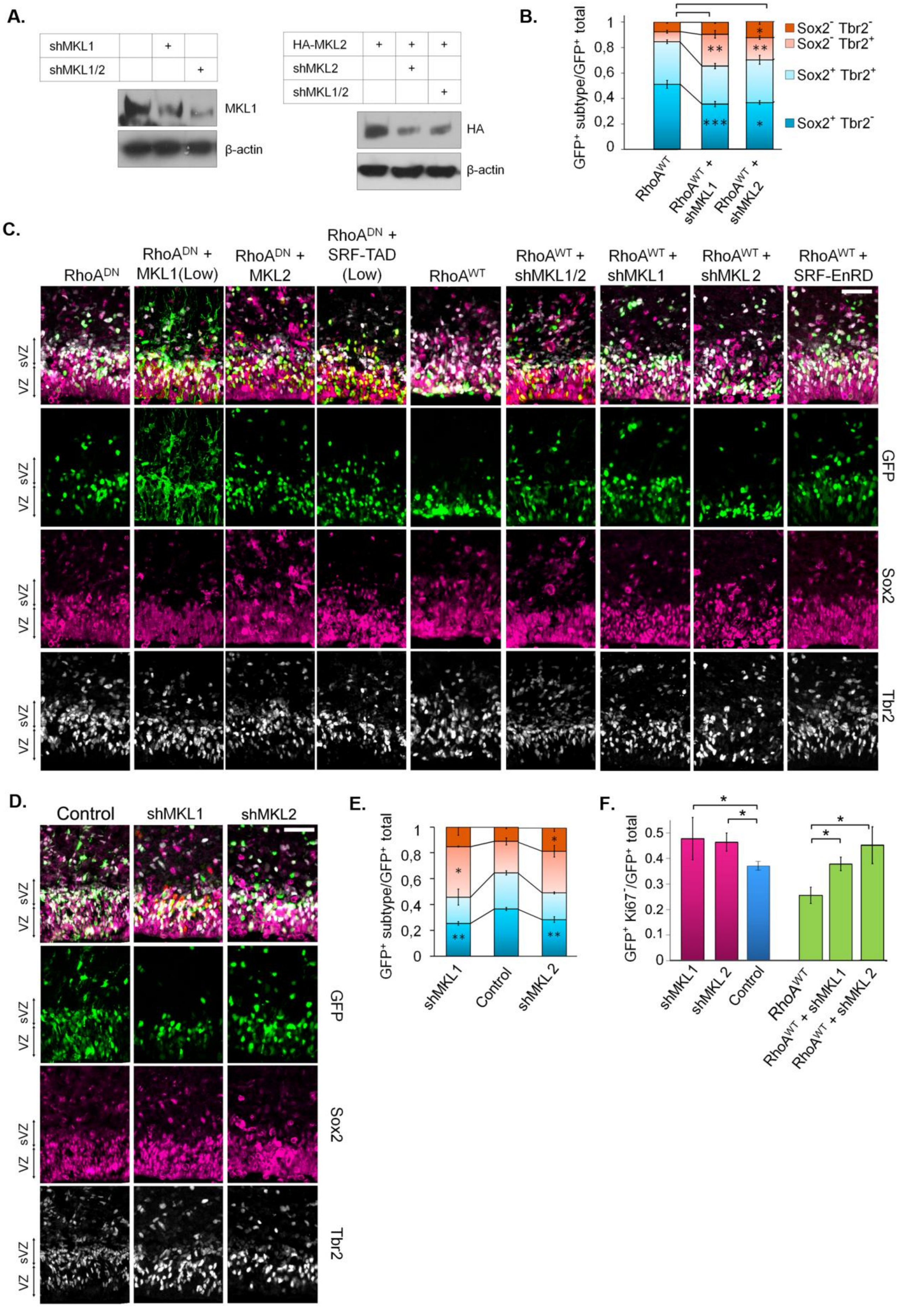

Fig. S2: The MKL/SRF complex regulates neurogenesis downstream of RhoA

(A) Efficiency of shMKL1, shMKL2 or a shRNA targeting both MKL1 and MKL2 (shMKL1/2). Neuro-2a cells were transfected for the expression of the different shRNAs and/or an HA-tagged MKL2 as indicated. Western blots were performed on cell lysates to reveal the endogenous MKL1 or the HA-tagged MKL2. **(B-F)** Embryonic brains were *in utero* electroporated at E15.5 and processed 23 hours later for immunohistological labelling. **(B,E)** Quantification of the $GFP^{+}Sox2^{+}Tbr2^{-}$ (RGCs), $GFP^{+}Sox2^{+}Tbr2^{+}$ (Committed BPs), $GFP^{+}Sox2^{-}Tbr2^{+}$ (BPs), $GFP^{+}Sox2^{-}Tbr2^{-}$ (neurons) cells. (**B**) Epistasis experiments are statistically tested against $RhoA^{WT}$. $RhoA^{WT}$ n=7 out of 6 IUE (n=7//6); $RhoA^{WT}$ + shMKL1 n=15//5; $RhoA^{WT}$ + shMKL2 n=5//3. (**E**) Control n=9//5; shMKL1 n=3//3; shMKL2 n=5//3. **(C,D)** Coronal sections of E16.5 cerebral cortices. Brains were in utero electroporated at E15.5 with the indicated plasmids along with NLS-GFP and stained for the indicated markers. **(F)** Quantification of $GFP^{+}Ki67^{-}$ cells (exit from cell cycle). Control n=12//9; $RhoA^{WT}$ n=9//7; shMKL1 n=6//3; shMKL2 n=4//3; $RhoA^{WT}$ + shMKL1 n=15//5; $RhoA^{WT}$ + shMKL2 n=4//3. Error bars, s.e.m., ***p<0.001, **p<0.01, *p<0.05, Scale bars: 50µm.

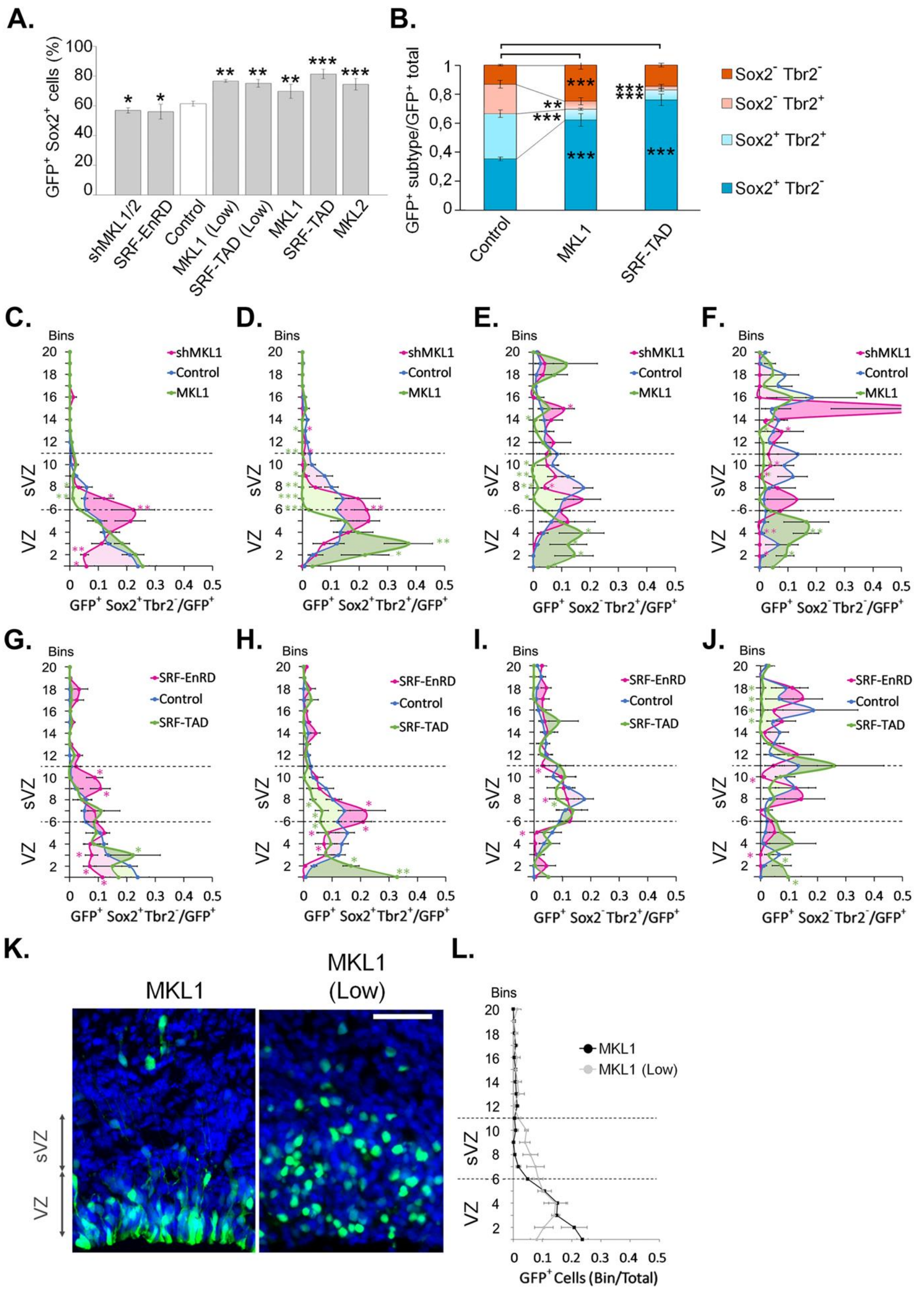

Fig. S3: MKL1 and SRF regulate progenitors differentiation and position

(A,B) SRF and MKL modulate progenitors differentiation. Brains were *in utero* electroporated at E15.5 and collected at E16.5. The graphics indicate the percentage of (**A**) GFP$^+$Sox2$^+$ cells or (**B**) GFP$^+$Sox2$^+$ Tbr2$^-$ (RGCs), GFP$^+$Sox2$^+$Tbr2$^+$ (Committed BPs), GFP$^+$Sox2$^-$Tbr2$^+$ (BPs), GFP$^+$Sox2$^-$Tbr2$^-$ (neurons) for brains electroporated with control, shMKL1/2, MKL1 at a low concentration (MKL1(Low)), MKL1, MKL2, SRF-EnRD, SRF-TAD at a low concentration (SRF-TAD(Low)), or SRF-TAD expression vectors. (**A**) Control n=19 out of 13 IUE (n=19//13); shMKL1/2 n=11//6; SRF-EnRD n=7//4; MKL1(Low) n=4//3; SRF-TAD(Low) n=8//3; MKL1 n=6//4; SRF-TAD n=5//3; MKL2 n=6//5; (**B**) Control n=9//5; MKL1 n=4//4; SRF-TAD n=4//3. **(C-J)** Radial distribution of the GFP$^+$Sox2$^+$Tbr2$^-$ (RGCs), GFP$^+$Sox2$^+$Tbr2$^+$ (Committed BPs), GFP$^+$Sox2$^-$Tbr2$^+$ (BPs), GFP$^+$Sox2$^-$Tbr2$^-$ (neurons) cells of brains electroporated with the expression plasmids for the indicated proteins at E15.5 and processed at E16.5. (**C-F**) Control n=9//5; shMKL1 n=6//3; MKL1 n=9//4; (**G-J**) Control n=9//5; SRF-EnRD n=8//3; SRF-TAD n=4//3. **(K,L)** Cell position and differentiation are

sensitive to MKL/SRF dosage. Coronal sections of E16.5 mice cerebral cortices electroporated with GFP expression plasmid and high or low concentrations of MKL1 expression vectors at E15.5, and stained for DAPI. The graphics indicate the percentage of cells in each bin of GFP+ cells for MKL1 expression vectors electroporated at E15.5, either with a high or low concentration and observed at E16.5. MKL1 n=12//6; MKL1(Low) n=5//3. Error bars, s.e.m., ***p<0.001, **p<0.01, *p<0.05, Scale bar: 50µm.

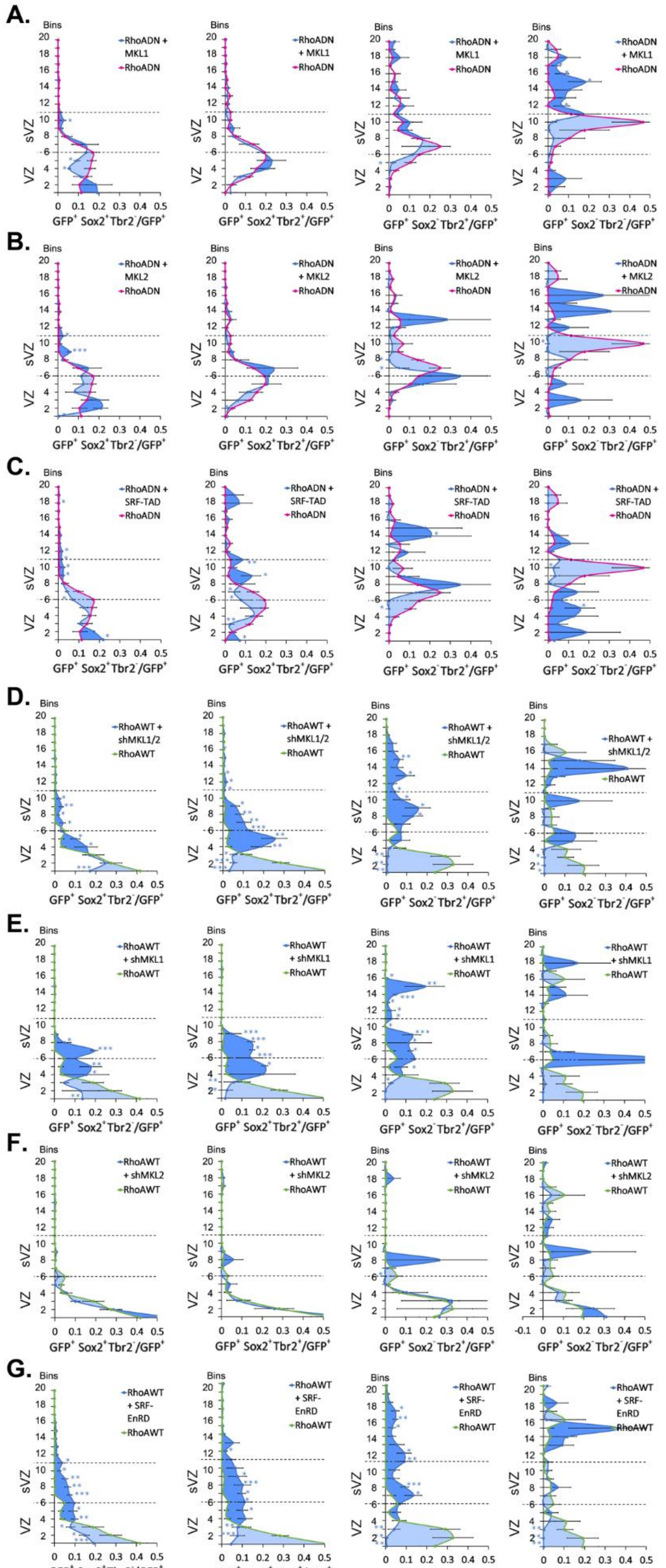

Fig. S4: The MKL/SRF complex regulates cell positioning downstream of RhoA.

Radial distribution of the $GFP^+Sox2^+Tbr2^-$ (RGCs), $GFP^+Sox2^+Tbr2^+$ (Committed BPs), $GFP^+Sox2^-Tbr2^+$ (BPs), $GFP^+Sox2^-Tbr2^-$ (neurons) cells of brains electroporated with the expression plasmids for the indicated proteins at E15.5 and processed at E16.5 for immunohistological labelling. $RhoA^{DN}$ n=6 out of 6 IUE (n=6//6); $RhoA^{DN}$ + MKL1 n=4//3; $RhoA^{DN}$ + MKL2 n=4//3; $RhoA^{DN}$ + SRF-TAD n=6//3; $RhoA^{WT}$ n=7//6; $RhoA^{WT}$ + shMKL1/2 n=5//3; $RhoA^{WT}$ + shMKL1 n=15//5; $RhoA^{WT}$ + shMKL2 n=5//3; $RhoA^{WT}$ + SRF-EnRD n=8//4; Error bars, s.e.m. ***$p<0.001$, **$p<0.01$, *$p<0.05$.

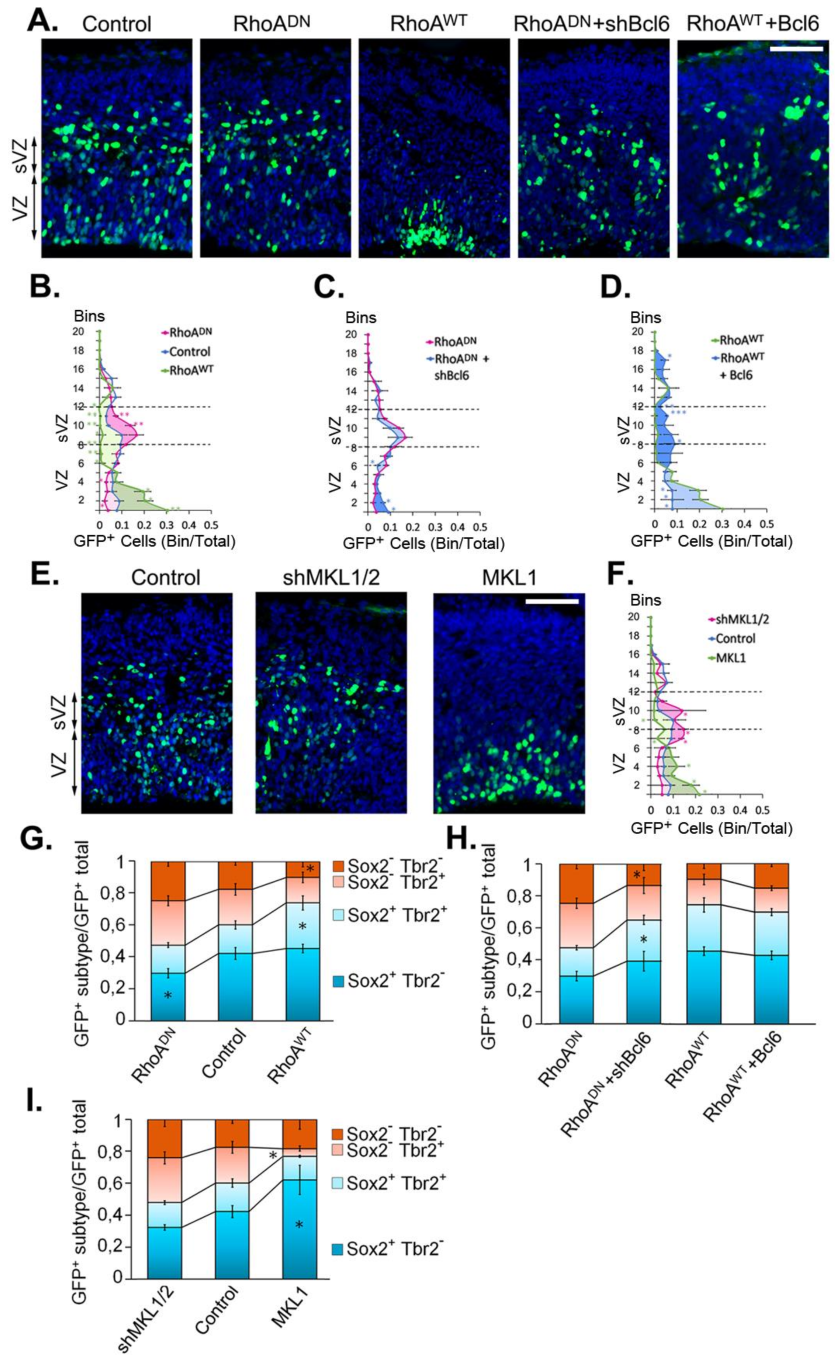

Fig. S5: The pathway is also active during earlier stages of neurogenesis.

(A-F) Coronal sections of mice cerebral cortices electroporated at E13 with either control, dominant-negative (RhoA^{DN}), wild type RhoA (RhoA^{WT}), shMKL1/2 or MKL1 expression vectors, co-electroporated with NLS-GFP and stained for DAPI 22 hours later. Cerebral walls from VZ to pia were subdivided into 20 bins. The graphics indicate the proportion of the GFP+ cells in each bin. Control n=9 out of 4 IUE (n=9//4); RhoA^{DN} n=6//3; RhoA^{WT} n=9//4; RhoADN+shBcl6 n=5//3; RhoAWT+Bcl6 n=4//3; shMKL1/2 n=4//3; MKL1 n=3//3; **(G-I)** Quantification of the triple staining GFP^{+}Sox2^{+} Tbr2^{-}, GFP^{+}Sox2^{+}Tbr2^{+}, GFP^{+}Sox2^{-}Tbr2^{+}, GFP^{+}Sox2^{-}Tbr2^{-} cells. Control n=6//4; RhoA^{DN} n=7//3; RhoA^{WT} n=7//4; shMKL1/2 n=4//3*; MKL1 n=3//3; RhoA^{DN}* + *shBcl6 n=5//3), RhoA^{WT}* + *Bcl6 n=3//3*. Error bars, s.e.m. ***p<0.001, **p<0.01, *p<0.05. Scale bar: 50µm.

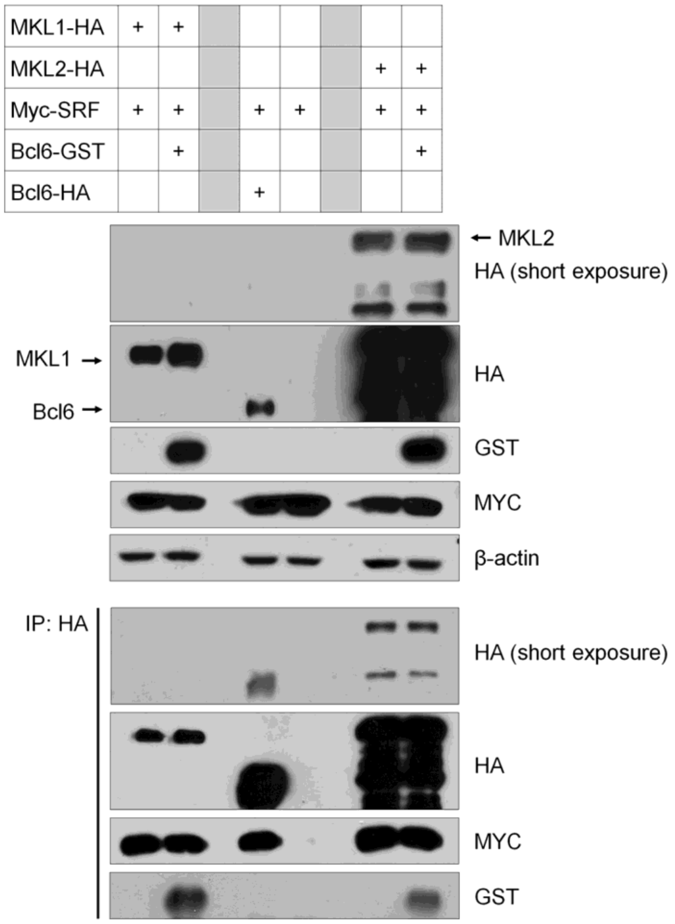

Fig. S6: Bcl6 physically interacts with SRF and can be found in a tripartite complex with SRF and MKL1 or MKL2.

Co-Immunoprecipitation assays were performed using tagged Bcl6, SRF, MKL1, and MKL2 expressed in HEK293T. 24h post transfection, cell lysates were analyzed directly or after pull-down with an HA antibody. Samples were immunoblotted with HA, Myc or GST antibodies.

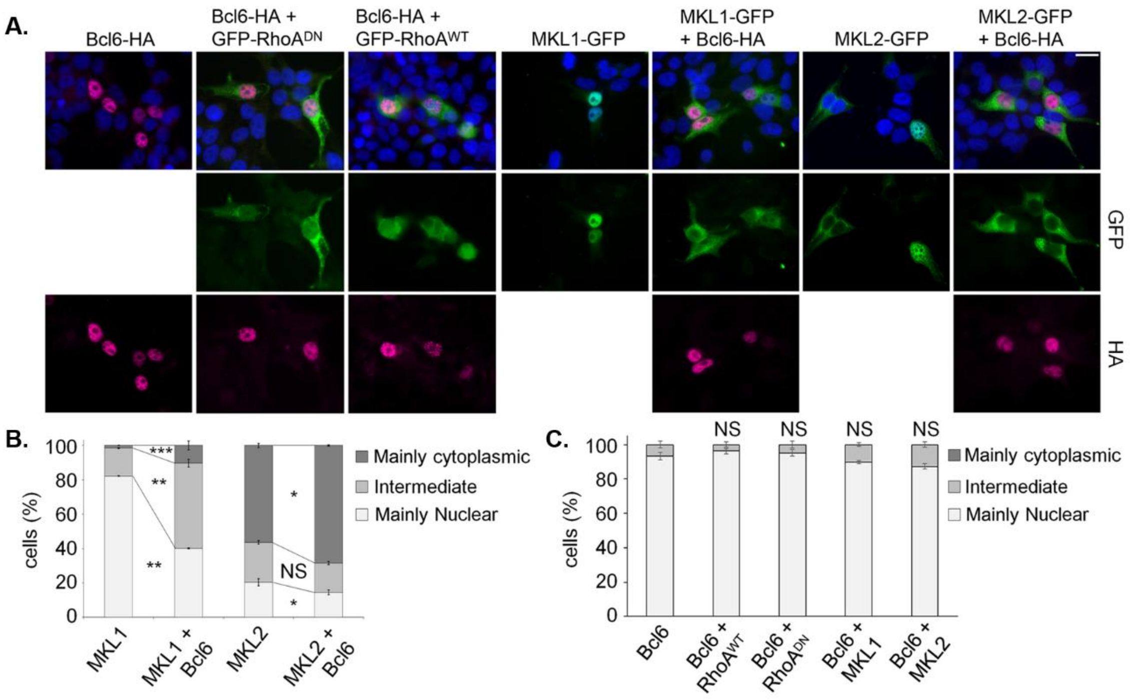

Fig. S7: Bcl6 partially displaced both MKL1 and MKL2 towards the cytoplasm, while the RhoA/MKL pathway did not affect Bcl6 predominant nuclear location

(A) Epifluorescence microscopic images of HeLa cells transfected with MKL1-GFP, MKL2-GFP, GFP-RhoA^{DN}, GFP-RhoA^{WT}, and/or Bcl6-HA in the indicated conditions, 24h post transfection. Cells were stained for HA when necessary, and all stained with DAPI. **(B,C)** Quantification of the essentially cytoplasmic (at least 60% of the signal in the cytoplasm), intermediate (fluorescence in the nucleus and the cytoplasm between 40% and 60%), or essentially nuclear (at least 60% of the signal in the nucleus) fluorescence localization for the indicated proteins. The tests were performed in Hela or HEK293T cells transfected for 24h with similar results. The fluorescence intensity was quantified using the RGB Profile Plot from ImageJ. The measurement of blue fluorescence, corresponding to DAPI, allowed us to delineate the nucleus from the cytoplasm. n ≥ 3; Error bars, s.e.m. ***p<0.001, **p<0.01, *p<0.05, NS, not significant; Scale bar: 50µm.

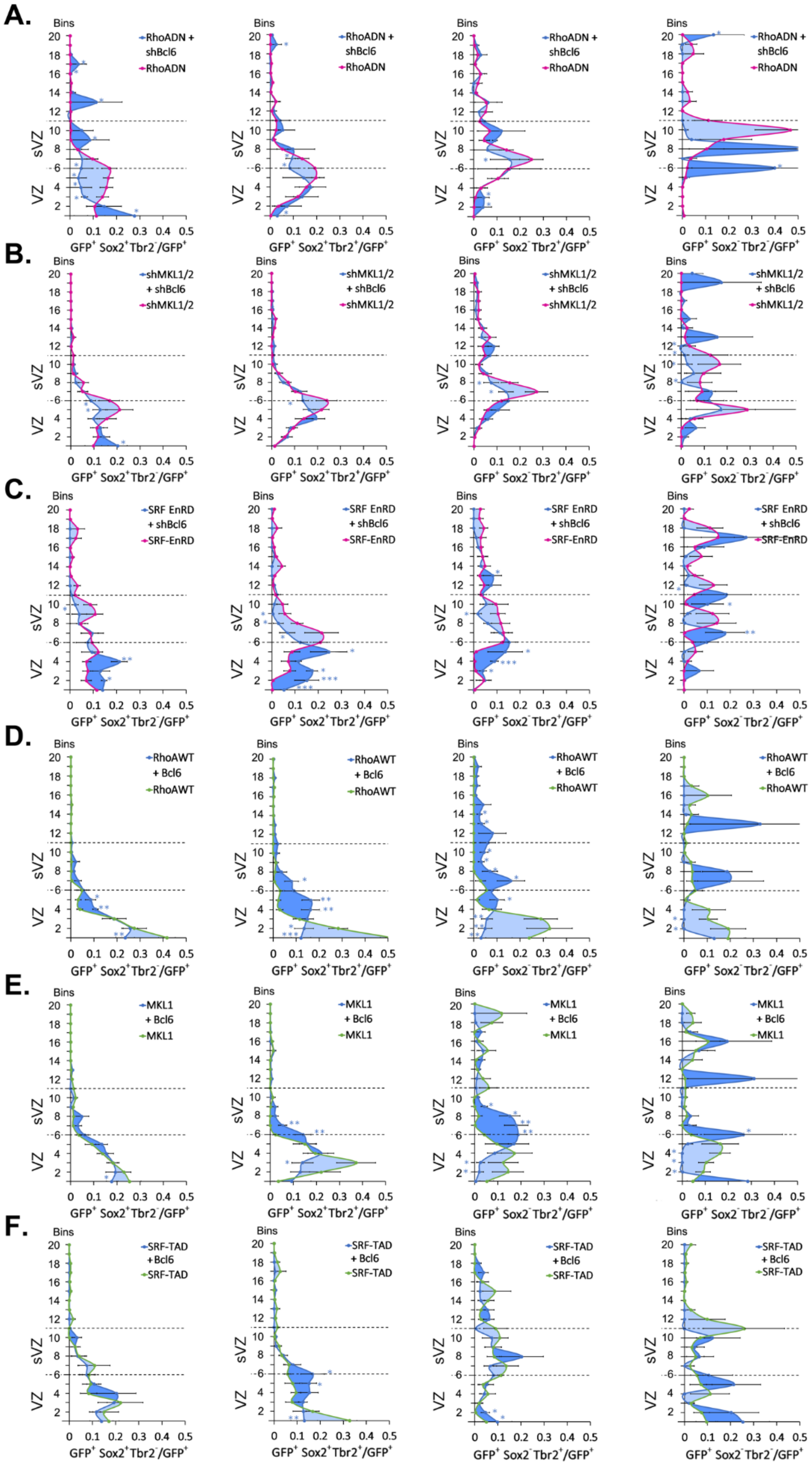

Fig. S8: Bcl6 opposes RhoA/MKL/SRF function on cell positioning *in vivo*

The graphics indicate the percentage of cells in each bin of GFP$^+$Sox2$^+$Tbr2$^-$, GFP$^+$Sox2$^+$Tbr2$^+$, GFP$^+$Sox2$^-$Tbr2$^+$, GFP$^+$Sox2$^-$Tbr2$^-$ for the indicated expression vectors electroporated at E15.5 and observed at E16.5. RhoA^{DN} n=8 out of 7 IUE (n=8//7); RhoA^{DN} + shBcl6 n=10//4; shMKL1/2 n=6//3; shMKL1/2 + shBcl6 n=4//3; SRF-EnRD n=8//3; SRF-EnRD + shBcl6 n=3//3; RhoA^{WT} n=7//6; RhoA^{WT} + Bcl6 n=7//5; MKL1

n=9//4; MKL1 + Bcl6 n=5//3; SRF-TAD n=4//3; SRF-TAD + Bcl6 n=4//3; Error bars, s.e.m. ***p<0.001, **p<0.01, *p<0.05.

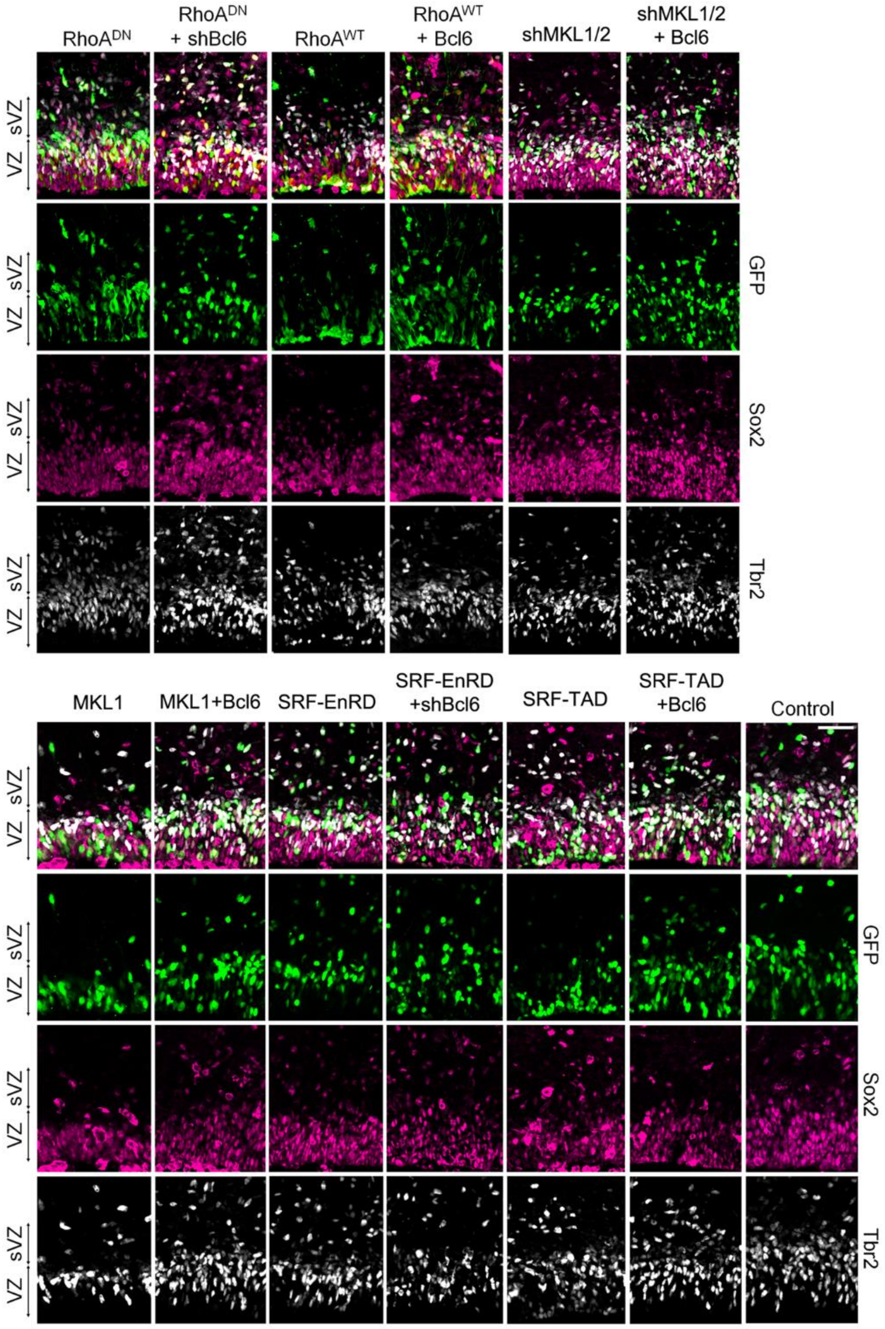

Fig. S9: Coronal sections of E16.5 cerebral cortices.

Brains were *in utero* electroporated at E15.5 with the indicated plasmids along with NLS-GFP and stained for the indicated markers 23 hours later. Scale bar: 50µm.

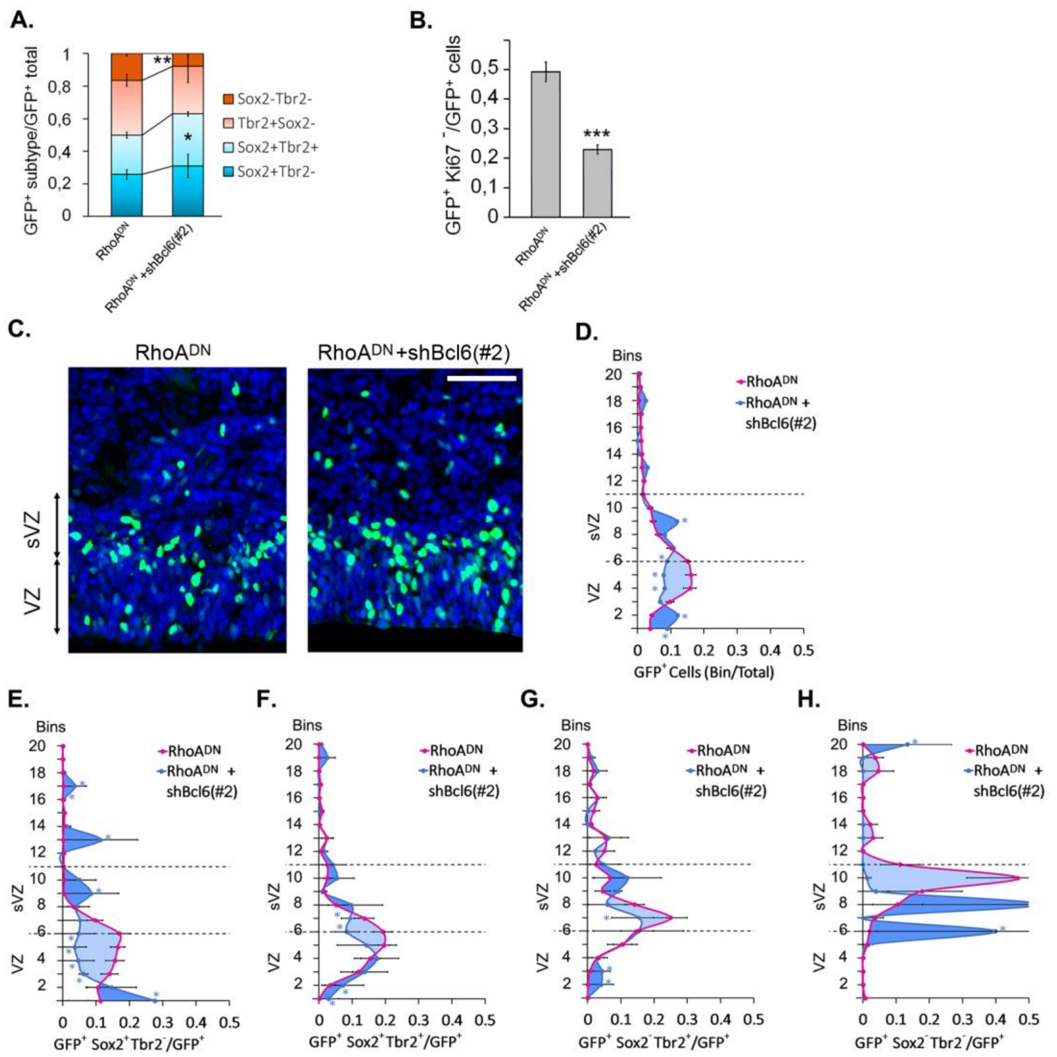

Fig. S10: Rescue experiments with a second shRNA targeting Bcl6

(A,B) Quantification of the GFP$^+$Sox2$^+$Tbr2$^-$, GFP$^+$Sox2$^+$Tbr2$^+$, GFP$^+$Sox2$^-$Tbr2$^+$, GFP$^+$Sox2$^-$Tbr2$^-$, GFP$^+$Ki67$^-$ cells. (**A**) RhoA^{DN} n=8 out of 7 IUE (n=8//7); RhoA^{DN} + shBcl6(#2) n=3//3; (**B**) RhoA^{DN} n=11//6; RhoA^{DN} + shBcl6(#2) n=3//3; (**C**) Coronal sections of E16.5 mice cerebral cortices electroporated with either dominant-negative (RhoA^{DN}), or RhoA^{DN} and shBcl6(#2) expression vectors at E15.5, co-electroporated with NLS-GFP and stained for DAPI. **(D-H)** Graphics of cerebral walls from electroporated brains, corresponding to the VZ to the upper part of the IZ, and subdivided into 20 bins. The graphics indicate the proportion of cells in each bin of (**D**) GFP$^+$ cells or (**E**) GFP$^+$Sox2$^+$ Tbr2$^-$ (RGCs), (**F**) GFP$^+$Sox2$^+$Tbr2$^+$ (Committed BPs), (**G**) GFP$^+$Sox2$^-$Tbr2$^+$ (BPs), and (**H**) GFP$^+$Sox2$^-$Tbr2$^-$ (neurons) RhoA^{DN} n=7 out of 6 IUE; RhoA^{DN} + shBcl6(#2) n=4 out of 3 IUE. Error bars, s.e.m. ***p<0.001, **p<0.01, *p<0.05, Scale bar: 50µm.

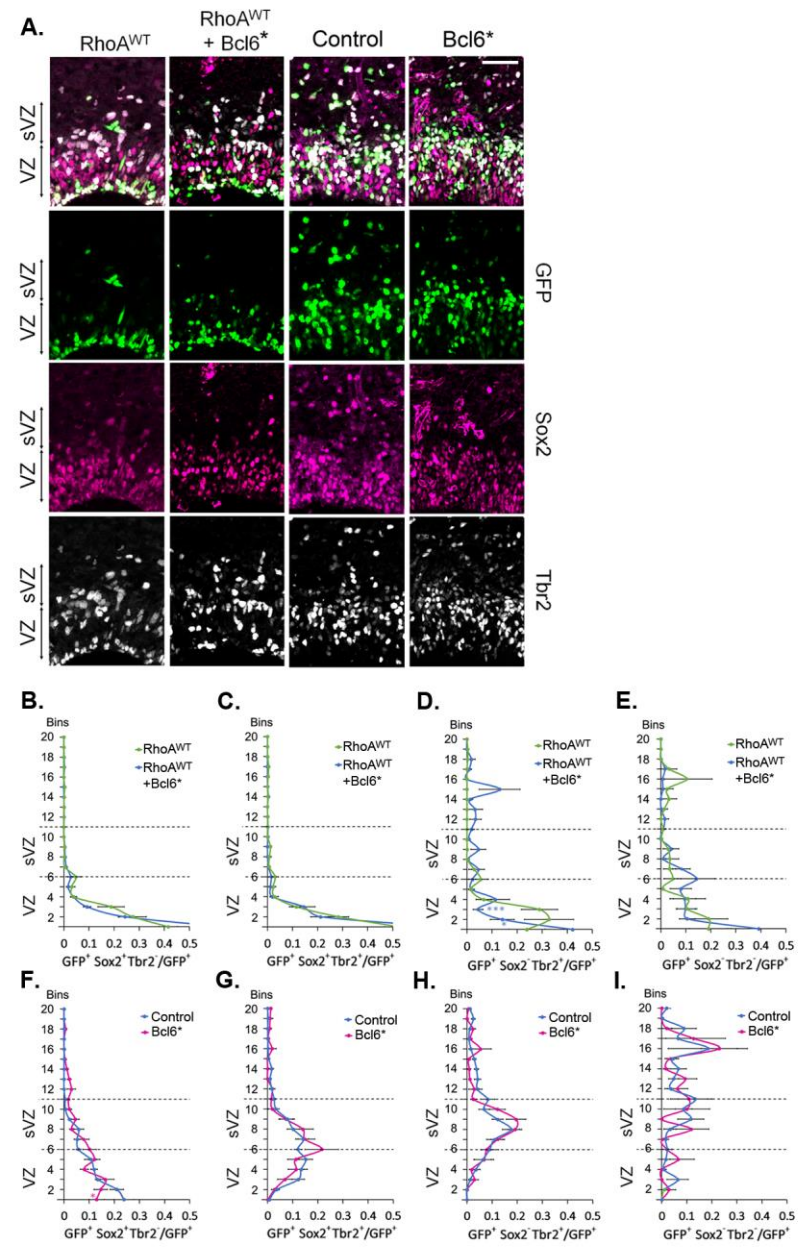

Fig. S11: a mutated version of Bcl6 unable to interact with SRF could not rescue the positional phenotypes induced by RhoA GoF

(A) Coronal sections of E16.5 mice cerebral cortices electroporated for the expression of the indicated protein at E15.5, co-electroporated with NLS-GFP and stained for Sox2 and Tbr2. **(B-I)** Graphics of cerebral walls from electroporated brains, corresponding to the VZ to the upper part of the IZ, and subdivided into 20 bins. The graphics indicate the proportion of cells in each bin of $GFP^{+}Sox2^{+}$ $Tbr2^{-}$ (RGCs), $GFP^{+}Sox2^{+}Tbr2^{+}$ (Committed BPs), $GFP^{+}Sox2^{-}Tbr2^{+}$ (BPs), and $GFP^{+}Sox2^{-}Tbr2^{-}$ (neurons) $RhoA^{WT}$ n=7 out of 6 IUE (n=7//6); $RhoA^{WT}$ + Bcl6* n=9//3, Control n=9//5; Bcl6* n=11//4; Error bars, s.e.m. ***p<0.001, **p<0.01, *p<0.05. Scale bar: 50µm.

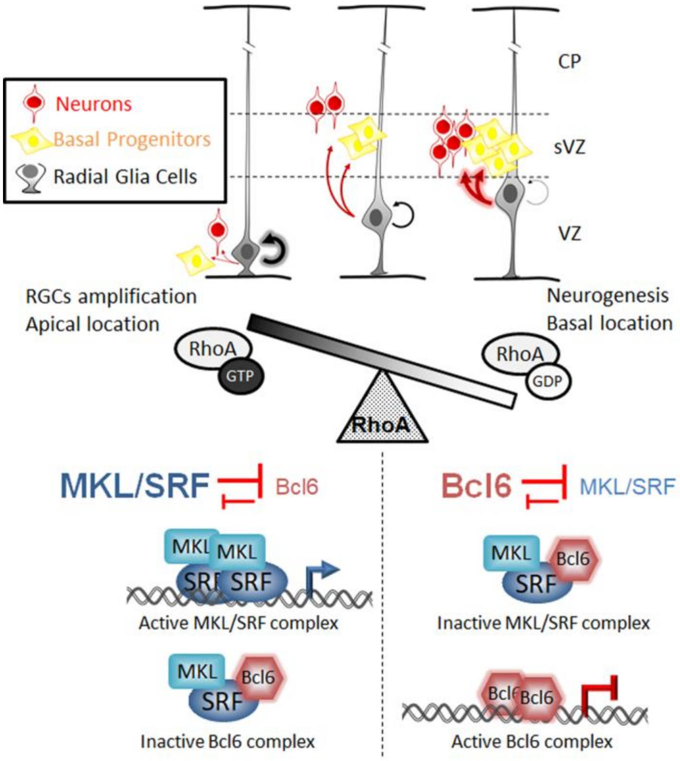

Fig. S12: Proposed model for the regulation of neocortical progenitors positioning and neurogenesis by the mutual repression of Bcl6 and the RhoA/MKL/SRF pathway.

During the development of the neocortex, the MKL/SRF complex maintains the neural progenitor pool size and regulates the position of Radial Glia Cells and Basal Progenitors under the control of RhoA. Bcl6 opposes these functions of RhoA/MKL/SRF through a reciprocal regulatory loop whereby Bcl6 and MKL/SRF antagonize each other's activity. Homodimerization, a prerequisite for the activity of both SRF and Bcl6, is inhibited by their direct physical interaction.

Movies 1 & 2: Time lapse videomicroscopy of control and RhoA-inhibited cells in organotypic brain slice cultures

Time lapse analysis of RGCs in the VZ. Brains were *in utero* electroporated at E15.5 for the expression of GFP (Movie 1) or GFP and RhoADN (movie 2). Brain slices were produced and processed for videomicroscopy 14 hours later using confocal microscopy. Frame are every 20 minutes. Compressed z-stacks spanning 90 µm of cortical depth. To facilitate tracking, green dots follow the movement of the nuclei of some RGCs with their apical process attached to the apical surface. Red dots follow the movement of cells attached to the apical surface and with their nuclei remaining at a basal position.

Table S1: Key resources

Key Resources Table				
**Reagent type (species) or resource**	**Designation**	**Source or reference**	**Identifiers**	**Additional information**
strain background (Escherichia coli)	One shot TOP10	Fisher Scientific	Cat #: C404010	Chemically Competent Cells
strain background (Escherichia coli)	NEB 10-beta	New England Biolabs	Cat#: C2987	Chemically Competent Cells
strain background (Mus Musculus)	CD1	Charles River Laboratories	022	
cell line (Homo sapiens)	HEK293T cells	ATCC	Ca#: CRL-3216, RRID:CVCL_0063	
cell line (Homo sapiens)	HeLa cells	ATCC	Cat# CRM-CCL-2, RRID:CVCL_0030	
cell line (Mus musculus)	Neuro-2a	ATCC	Cat# CCL-131, RRID:CVCL_0470	
Antibody	Anti-HA.11 clone 16B12 (Mouse monoclonal)	Biolegend	Cat# 901505, RRID: RRID:AB_2565023	
Antibody	rabbit anti-HA.11	Biolegend	Cat # : 902301, RRID:AB_2565018	
Antibody	Anti-Myc (Rabbit polyclonal)	Cell Signaling Technology	Cat# 2272, RRID: AB_10692100	
Antibody	Anti-Myc-tag clone 9B11 (Mouse monoclonal)	Cell Signaling Technology	Cat# 2276, RRID: AB_331783	
Antibody	Anti-β-actin (Mouse monoclonal)	Fisher Scientific	Cat# MA5-15739, RRID: AB_10979409	
Antibody	Anti-GFP (Rabbit polyclonal)	Fisher Scientific	Cat# A-11122, RRID: AB_221569	
Antibody	Anti-GFP (Chicken polyclonal)	Aves	Cat# GFP-1020, RRID:AB_10000240	
Antibody	Anti-Ki67 (Mouse monoclonal)	BD Biosciences	Cat# 556003, RRID: AB_396287	
Antibody	Phospho-Histone H3 (Ser10) (Rabbit polyclonal)	Thermo Fisher	Cat# PA5-17869, RRID:AB_10984484	
Antibody	Anti- Sox2 (L1D6A2) (Mouse monoclonal)	Cell Signaling Technology	Cat# 4900, RRID: AB_10560516	
Antibody	rabbit anti-Sox2	Millipore	Cat # : AB5603, RRID:AB_2286686	
Antibody	Anti-Tbr2 (Rabbit polyclonal)	Abcam	Cat# ab23345, RRID: AB_778267	
Antibody	Anti-Satb2 (mouse	Abcam	Cat# ab51502 RRID:AB_882455	
Antibody	mouse anti-Nestin	Millipore	Cat # : MAB353, RRID:AB_94911	
Antibody	Anti-GST	Sigma	Cat # : G1160, RRID:AB_259845	
Antibody	rabbit anti-α-Catenin	Sigma	Cat # : C2081, RRID:AB_476830	

Antibody	rabbit anti-ZO.1	Invitrogen	Cat # : 61-7300, RRID:AB_138452	
Antibody	Mouse anti-MKL1	Santa Cruz	Cat# sc-390324, RRID:AB_2891290	
Antibody	Anti-mouse IgG, HRP-linked Antibody (horse)	Cell Signaling Technology	Cat# 7076, RRID:AB_330924	
Antibody	Anti-rabbit IgG, HRP-linked Antibody (goat polyclonal)	Cell Signaling Technology	Cat# 7074, RRID:AB_2099233	
Antibody	Anti-Mouse IgG (H+L) Cross-Adsorbed Secondary Antibody, Alexa Fluor 488 (Goat Polyclonal)	invitrogen	Cat# A-11001, RRID: AB_2534069	
Antibody	Anti-Rabbit IgG (H+L) Antibody, Alexa Fluor 488 Conjugated (Goat polyclonal)	invitrogen	Cat# A-11008, RRID: AB_143165	
Antibody	Anti-Mouse IgG (H+L) Antibody, Alexa Fluor 568 Conjugated (goat polyclonal)	invitrogen	Cat# A-11004, RRID: AB_2534072	
Antibody	Anti-Rabbit IgG (H+L) Cross-Adsorbed Secondary Antibody, Alexa Fluor 568 (Goat polyclonal)	invitrogen	Cat# A-11011, RRID: AB_143157	
Antibody	Anti-Mouse IgG (H+L) Cross-Adsorbed Secondary Antibody, Alexa Fluor 647 (Goat polyclonal)	invitrogen	Cat# A-21235, RRID: AB_2535804	
Antibody	Anti-Rabbit IgG (H+L) Cross-Adsorbed Secondary Antibody, Alexa Fluor 647 (Goat polyclonal)	invitrogen	Cat# A-21244, RRID: AB_2535812	
Antibody	Anti-Chicken IgY (H+L) Cross-Adsorbed Secondary Antibody, Alexa Fluor 488 (Goat Polyclonal)	Invitrogen	Cat#: A32931, RRID:AB_2762843	
Recombinant DNA reagent	pCAG:GFP	Addgene	RRID:Addgene_11 150	
recombinant DNA reagent	pCAG:NLS-GFP	F. Kubo, National Institute of Genetics	N/A	
Recombinant DNA reagent	pEBG	Addgene	RRID: Addgene_22227	
Recombinant DNA reagent	UBQ10:sXVE:(MCS)-S11-DI-GFP1–9	Addgene	RRID: Addgene_108260	S11 and GFP1-9 subcloned in pCAG vector
Recombinant DNA reagent	UBQ10-sXVE-S10-(MCS)-3xHA	Addgene	RRID: Addgene_108178	S10 subcloned in pCAG vector
Recombinant DNA reagent	pCAG:Myc-RhoA	This paper	N/A	
Recombinant DNA reagent	pCAG:Myc-RhoA(T19N)	This paper	N/A	
Recombinant DNA reagent	pCAG:MKL1-HA	This paper	N/A	
Recombinant DNA reagent	pCAG:MKL1-GFP	This paper	N/A	

Recombinant DNA reagent	pCAG:S10-MKL1	This paper	N/A	
Recombinant DNA reagent	pCAG:S10-MKL1ΔLZ	This paper	N/A	
Recombinant DNA reagent	pCAG:MKL2-HA	This paper	N/A	
Recombinant DNA reagent	pCAG:S10-MKL2	This paper	N/A	
Recombinant DNA reagent	pCAG:Myc-SRF	This paper	N/A	
Recombinant DNA reagent	pCAG:S11-SRF	This paper	N/A	
Recombinant DNA reagent	pCAG:SRF-TAD	This paper	N/A	
Recombinant DNA reagent	pCAG:SRF-EnRD	This paper	N/A	
Recombinant DNA reagent	pCAGIG:Bcl6	Pierre Vanderhaeghen	N/A	Bcl6 subcloned into pCAG vector
Recombinant DNA reagent	pCAG:Bcl6-HA	This paper	N/A	
Recombinant DNA reagent	pCAG:Bcl6-GST	This paper	N/A	
Recombinant DNA reagent	pCAG:S11-Bcl6	This paper	N/A	
Recombinant DNA reagent	pCAG:Bcl6(C121F;P421L)-HA	This paper	N/A	
Recombinant DNA reagent	pCAG:S11-Bcl6(C121F;P421L)	This paper	N/A	
Recombinant DNA reagent	pGL4:SRF-RE-Luc	Sigma	Cat #: E1350	
Recombinant DNA reagent	pBCl6(BS)-Luc	This paper	N/A	
Recombinant DNA reagent	tFucci(SA)5	Addgene	RRID: Addgene_153520	
Recombinant DNA reagent	pSCV2:shBcl6 (#1)	Pierre Vanderhaeghen	N/A	Target sequence: 5’-gacacggatctgagaatct-3’
Recombinant DNA reagent	pSCV3:shBcl6 (#1)	This paper	N/A	Deletion of VenusFP coding sequence
Recombinant DNA reagent	pSCV3:shBcl6 (#2)	This paper	N/A	Target sequence: 5’-tgatgttcttctcaaccttaa-3’
Recombinant DNA reagent	pSCV2:control	Pierre Vanderhaeghen	N/A	Target sequence: 5’-actaccgttgttataggtg-3’
Recombinant DNA reagent	pSCV3:control	This paper	N/A	Deletion of VenusFP coding sequence
Recombinant DNA reagent	pLKO.1.shMKL1/2	Addgene	RRID: Addgene_27161	Target sequence: 5’-CATGGAGCTGGTGGAGAAGAA-3’
Recombinant DNA reagent	pSCV3:shMKL1	This paper	N/A	Target sequence: 5’-GGTAGCAGACAGTTCCTCC-3’

Recombinant DNA reagent	pSCV3:shMKL2	This paper	N/A	Target sequence: 5'-GCCATCCCAAGAATC CAAA-3'
chemical compound, drug	Protease inhibitor cocktail	Roche	Cat# 05056489001	
chemical compound, drug	Phosphatase inhibitor cocktail	Roche	Cat #: A32957	
chemical compound, drug	B27	invitrogen	Cat #: 17504-044	
Chemical compound, drug	Penicillin-streptomycin	Gibco	Cat #: 11548876	
Chemical compound, drug	EdU	Abcam	Cat #: ab146186	
Chemical compound, drug	Sulfo-Cyanine3 azide	Lumiprobe	Cat # : B1330	
commercial kit	Plasmid DNA Purification Mini Kit	Intron Biotechnology	Cat# 17098	
commercial kit	Quick Gel extraction Kit	Fisher Scientific	Cat# K2100-12	
commercial kit	HiPure Plasmid Maxiprep Kit	Fisher Scientific	Cat# K2100-07	
software, algorithm	Image J	NIH	N/A	
software, algorithm	QuPath		N/A	
software, algorithm	Zen Lite	Zeiss	N/A	
other	DAPI staining	Sigma	Cat #: D9542	
other	PolyJet™ In Vitro DNA Transfection Reagent	Signagen	Cat #: SL100688	
Other	Dynabeads protein A	invitrogen	Cat #: 10001D	
Other	Dynabeads protein G	İnvitrogen	Cat #: 1003D	
other	Super signal West Pico PLUS chemuluminescent substrate	Fisher Scientific	Cat #: 34578	
other	O.C.T.	Sakura	Cat # 4583	
other	DMEM-F-12	Gibco	Cat #: 21331-020	
other	DMEM, high glucose	Gibco	Cat #: 41965-039	
other	CL-X Posure film	Fisher Scientific	Cat #: 34091	
